# Systematic review of control groups in nutrition education intervention research

**DOI:** 10.1186/s12966-017-0546-3

**Published:** 2017-07-11

**Authors:** Carol Byrd-Bredbenner, FanFan Wu, Kim Spaccarotella, Virginia Quick, Jennifer Martin-Biggers, Yingting Zhang

**Affiliations:** 1 Rutgers, The State University of New Jersey, 26 Nichol Avenue, New Brunswick, NJ 08901 USA; 2 Kean University, 1000 Morris Ave, Union, NJ 07083 USA

**Keywords:** Research design, Control group, Experimental group, Systematic review, Nutrition education

## Abstract

**Background:**

Well-designed research trials are critical for determining the efficacy and effectiveness of nutrition education interventions. To determine whether behavioral and/or cognition changes can be attributed to an intervention, the experimental design must include a control or comparison condition against which outcomes from the experimental group can be compared. Despite the impact different types of control groups can have on study outcomes, the treatment provided to participants in the control condition has received limited attention in the literature.

**Methods:**

A systematic review of control groups in nutrition education interventions was conducted to better understand how control conditions are described in peer-reviewed journal articles compared with experimental conditions. To be included in the systematic review, articles had to be indexed in CINAHL, PubMed, PsycINFO, WoS, and/or ERIC and report primary research findings of controlled nutrition education intervention trials conducted in the United States with free-living consumer populations and published in English between January 2005 and December 2015. Key elements extracted during data collection included treatment provided to the experimental and control groups (e.g., overall intervention content, tailoring methods, delivery mode, format, duration, setting, and session descriptions, and procedures for standardizing, fidelity of implementation, and blinding); rationale for control group type selected; sample size and attrition; and theoretical foundation.

**Results:**

The search yielded 43 publications; about one-third of these had an inactive control condition, which is considered a weak study design. Nearly two-thirds of reviewed studies had an active control condition considered a stronger research design; however, many failed to report one or more key elements of the intervention, especially for the control condition. None of the experimental and control group treatments were sufficiently detailed to permit replication of the nutrition education interventions studied.

**Conclusions:**

Findings advocate for improved intervention study design and more complete reporting of nutrition education interventions.

**Electronic supplementary material:**

The online version of this article (doi:10.1186/s12966-017-0546-3) contains supplementary material, which is available to authorized users.

## Background

A major goal of nutrition education research is to elucidate factors that enable individuals to improve diet-related behaviors and/or cognitions associated with better health and greater longevity. These factors can then be incorporated in educational and health promotion interventions which, in turn, can be evaluated to determine whether the intervention effects change behaviors and/or cognitions among those assigned to the intervention vs. those in a control condition.

Well-designed research trials are critical for determining the efficacy and effectiveness of new interventions [1]. The basic components of educational research intervention trials include experimental variables, such as a novel curriculum; strong, measurable research questions or hypotheses; valid and reliable instruments for documenting change in behavior and/or cognitions; a strong data analysis plan; and an experimental design that minimizes threats to internal validity. To determine whether behavioral and/or cognition changes can be attributed to the intervention, the experimental design must include a control or comparison condition against which outcomes from the experimental group can be compared [2–5]. The randomized controlled trial (RCT) is typically considered the “gold standard” for ascertaining intervention efficacy and effectiveness [2].

Experts emphasize that to robustly minimize biases and variability of factors that may influence intervention trial outcomes, the control and experimental conditions must: 1) contain randomly assigned participants; 2) occur simultaneously to ensure both conditions experience the same history (i.e., external events, such as political change, natural disasters, scientific discoveries) and maturation (i.e., internal events, such as physical growth, memory decline with aging); 3) be structurally equivalent on as many non-specific factors as possible (i.e., factors other than the “active” ingredients in the experimental condition, such as participant time commitment, format and timeline of activities and data collection, and extent of attention and support from research staff) [5]; and 4) offer equal value, attractiveness, credibility, and outcome expectations to keep participants blind to their condition assignment and thereby avoid novelty effects, differential dropout rates, disappointment arising from assignment to the control group, and/or efforts by control group participants to seek an alternate source of the treatment offered to the experimental group [1, 3, 4, 6–16]. The control condition also must not modify the intervention’s specific factors (i.e., behavior and/or cognitions targeted in the experimental condition) [4, 7].

To reduce the risk of a Type 1 error (acceptance of an ineffective intervention) [1, 9, 17], treatment received by control condition participants should differ from those in the experimental condition only in the non-receipt of the “active ingredient” of the intervention hypothesized to affect study outcomes [4, 6]. Rigorous control of non-specific factors, however, tends to increase intervention research costs because a plausible control intervention must be developed and implemented. Additionally, as the stringency of control exerted over non-specific factors increases, the risk of understating the effectiveness of the intervention rises because effect size is inversely associated with rigor of non-specific factor control [9, 17–19]. Therefore, to demonstrate statistically meaningful differences, larger sample sizes are needed to avoid Type 2 errors (failure to recognize an intervention is effective) and detect treatment effects when the control and experimental group treatments are structurally equivalent than when a less equivalent control treatment is used [1, 9, 17].

A key challenge to nutrition education researchers is selecting a suitable treatment for the control condition that is congruent with the research question, study resources, availability of standard treatment/usual care, and ethical considerations [7, 9, 10, 12, 20, 21]. Control condition participants may receive treatment ranging from nothing at all to extensive treatment in an alternate “active” control condition unrelated to the experimental condition. As indicated in Table 1, the type of control condition selected can have important effects on study resources, participants, internal validity, and outcomes. For instance, resource investment in the treatment for the control condition can range from zero for the inactive control to considerable for active control. Ethical issues may be more highly problematic in inactive control conditions when participants in need of the intervention are denied treatment, but ethical issues are lessened when a standard or usual treatment can be offered. Preventing disappointed control group participants from seeking alternate sources of the treatment may not be possible, which weakens internal validity and undermines a true evaluation of the intervention’s effect. Even in active control conditions where participants receive a contemporaneous intervention equal to the treatment condition in all aspects, except the “active ingredient”, researchers may inadvertently treat control participants differently. Those delivering the intervention (e.g., research staff, educators) also may dislike being in the control condition [22] and seek opportunities to provide participants with treatment like that being given to the experimental group.

**Table 1:** Control Condition Treatments

Control Condition Treatment	Pros (+) and Cons (−)
Inactive Control: Control group receives no comparison treatment at all during the study or receives treatment after the study ends.	(+) No resource input for control condition development.^a^ (+) Increased likelihood of yielding large effect size because least likely to change targeted cognitions or behaviors or these may worsen without treatment [1]. (+) Can help identify potential adverse effects of intervention [1]. (+) May be useful for pilot testing new interventions [1]. (−) Potential to overstate outcome of intervention because nearly all interventions are more effective in changing outcomes than simple passage of time [6, 85]. (−) Increased risk of control group refusal to participate. (−) Increased risk of attrition and/or seeking alternate source of treatment during the waiting period [10]. (−) Generally considered a weak design [7, 17].
No Treatment Control: Control participants receive no treatment.	Additional Points: (−) Ethical issues when depriving a group in need of intervention of help when a suitable standard treatment/usual care is available; ethical problem lessens when no immediate risks (e.g., disease treatment) [20, 90]. (−) Vulnerable to treatment fidelity issues (temptation of research staff/clinicians to offer some treatment to needy participant) [1].
Wait-list (delayed treatment) Control: Closely related to no treatment control; control participants wait until the study concludes to receive treatment. During waiting period, wait list control participants may receive standard treatment/usual care which may impact study outcomes.	Additional Points: (+) No additional input for control condition development, but implementation costs must be considered (+) All participants receive the “active ingredient” treatment. (−) Ethical issues lessened unless control group is in immediate need of treatment and available standard treatment/usual care not provided.
Active Control: Control group receives a different treatment contemporaneously with the experimental group.[4, 17]	(+) Considered a strong design [7]. (+) If control group is given a bona fide treatment, possibility of ethical issues diminished. (+) Controlling non-specific treatment effects (e.g., participant burden, activity, and data collection format and scheduling, attention from researchers) [85] minimizes threats to internal validity and permits effects of the intervention to be more accurately attributed to the “active ingredient” hypothesized to affect the dependent variables [7]. (−) Creating a credible control treatment that is equally preferred by participants is difficult [20]. (−) Detrimental effects may occur if action control treatments lead to inaccurate conclusions about their personal health or other conditions and/or lack of action to improve a health or other condition. See Pagoto et al. [91] for more detailed discussion.
Usual or Standard Treatment: Control participants receive a treatment that is typically offered.	Additional Points: (+) Limited additional resource input for control condition development. (+) Provides opportunity to investigate whether new intervention is superior to existing treatment (−) Non-specific treatment effects likely different from intervention (e.g., differs in frequency of contact, type of intervention [e.g., passive vs active], time commitment, and/or provider qualifications, experience, and/or researcher/clinician allegiance to the protocol) [6]. For instance, if the experimental condition requires greater effort, experimental group completers likely will be more motivated than control participants and confound results [6]. (−) “Usual” treatment interventions often do not exist in nutrition education, negating this as an option. (−) “Usual” treatment intervention components often insufficiently described (e.g., in peer-reviewed articles or implementation manuals) to permit comparison by external reviewers [1, 6, 25]. (−) Often no verification of fidelity of usual treatment to protocol implementation (e.g., process evaluation, manual, or oversight of providers) [1]. (−) Lack of equipoise (sincere uncertainty of whether intervention will be beneficial over usual practices) may affect research staff interactions with participants [1] (detailed implementation manuals, frequent process evaluation, and strong supervision can mitigate this) [1]. (−) Research staff personality differences and variations (even inadvertently) affect their behavior toward and expectations of control vs. experimental participants [6]. (−) Comparing “usual” practices to experimental condition is reasonable only if experimental participants are blind to the novelty of the experimental condition [2].
Alternative Active Treatment: Control group receives an alternative treatment equal in non-specific treatment effects (e.g., participant burden, activity, and data collection format and scheduling, attention from researchers) to the experimental group and differs only in the non-receipt of the “active ingredient” of the intervention hypothesized to affect the dependent variables (e.g., only the subject matter content of the intervention differs). [4, 6]	Additional Points: (+) Controls for non-specific treatment effects enhances ability to ascribe efficacy to the experimental treatment [7]. (−) Control treatment components often insufficiently described (e.g., in peer-reviewed articles or implementation manuals) to permit comparison by external reviewers [1, 6]. (−) Often no verification of fidelity to protocol implementation (e.g., process evaluation, manual, or oversight of providers) for either experimental or control groups [1]. (−) Research staff personality differences and variations (even inadvertently) affect their behavior toward and expectations of control vs. experimental participants [6]. (−) Comparing control and experimental condition is reasonable only if both are blind to treatment group assignment [2, 92]. (−) Additional resource input for control condition development; using alternative active treatment when the effect of attention on participant outcome is unknown may be an unnecessary expense. (−) Rigorous control of non-specific treatment tends to contribute to study effects (i.e., control participant improvement), thus larger sample sizes or an increased risk of Type 1 error (e.g., p-level set higher than typical <0.05) is needed to prevent erroneously rejecting effective interventions as ineffective and to detect potentially small yet clinically important effect sizes [1, 9, 13, 17, 24].
Dismantling (or Additive) Component Attention Control: Typically used with a multi-part intervention where the individual parts are separated to identify which are most salient to the outcomes (often with the goal of increasing cost-effectiveness by paring down intervention parts).[7] Example: study of the effectiveness of a self-instructional guide accompanied by telephone counseling compared to the guide alone.	Additional Points: (+) Method is well suited if “usual” care is effective and desire is to improve on it; also overcomes ethical issue of denying treatment to those in need [93]. (−) Adequate sample size needed for each part of the multi-part intervention [1]. (−) Outcomes may be confounded if effect is due to differing exposure levels rather than the added component itself [24]. (−) Lower statistical power if added parts have small effect compared to existing intervention [7]. (−) Lack of equipoise (genuine uncertainty of whether individual intervention parts will be beneficial alone and/or better than usual practices) may affect research staff interactions with participants[1] (detailed implementation manuals, frequent process evaluation, and strong supervision can mitigate this) [1].

^a^Resources include time investment by participant and/or researcher, money, and research staff expertise

Clearly, the efficacy and “effectiveness of the experimental condition inherently depends as much on the control condition as on the experimental condition” [1],p.276. Despite the impact different types of control groups can have on study outcomes [23], the treatment provided to participants in the control condition has received limited attention in the literature [1, 7, 12, 17, 20, 24–26] and sometimes is not even described in research designs [27, 28]; yet in the words of Mohr et al. with regard to psychological interventions, “inappropriate control conditions can overestimate the effectiveness of a treatment, or kill off a potentially useful treatment” [1],p.283. Thus, a systematic review of control groups in nutrition education interventions was conducted with the goal of better understanding how control conditions are described in peer-reviewed primary outcomes journal articles in comparison with experimental conditions. An additional goal of this investigation is to open discussions among colleagues as to how best to improve reporting of control and experimental condition treatments in intervention evaluation studies to facilitate advancement of the field.

## Methods

A systematic literature search was conducted after review of guidance from the Nutrition Education Systematic Review Project [29]. The study team then identified databases to use in the systematic review, search terms, and inclusion and exclusion criteria.

### Search strategies

Search strategies were formulated according to the PRISMA guidelines [30]. Subject headings or search terms unique to each database were identified and searched in combination with keywords derived from the major concepts of “nutrition education intervention” and “control groups” or “study design”. Table 2 shows the final search strategy for the selected databases (i.e., CINAHL, PubMed, PsycINFO, WoS, and ERIC). Searches were conducted in winter 2016.

**Table 2:** Search strategies for databases searched^a^

Databases	Search Strategies ^a^
CINAHL via EBSCO	#1 (SU nutrition n2 education AND Behavior) OR SU nutrition education OR (nutrition AND Instruction) OR TX “Nutrition Instruction” AND #2 SU control group OR research design
PubMed	#1 “Nutritional Sciences/education” [MeSH] OR (Nutrition AND (“Health Knowledge, Attitudes, Practice” [MeSH] OR “Health Education/methods” [MeSH] OR “Health Behavior” [MeSH] OR “Health Promotion/methods” [MeSH])) OR (Food AND Nutrition AND Education) AND #2 “Control Groups” [MeSH] OR “Research Design” [MeSH] OR “Randomized Controlled Trial as Topic” [MeSH] OR “Non-Randomized Controlled Trial as Topic” [MeSH]
PsycINFO via OvidSP	#1 (exp Nutrition/AND exp. Education/) OR exp. Health Education/OR Nutrition Instruction.mp. OR (exp Nutrition/AND exp. Intervention/) OR (exp Nutrition/AND (exp Education OR exp. Health Behavior/OR exp. Health Promotion/OR exp. Health Knowledge/)) AND #2 exp. Experiment Controls/OR exp. Experimental Design/OR Randomized Controlled Trial.mp.
Web of Science	#1 “Nutrition Education” OR “Nutrition Instruction” OR “Nutrition Intervention” AND #2 “Control Groups” OR “Research Design” OR “Quasi experimental Design”
ERIC via EBSCO	#1 “Nutrition Instruction” OR (“Nutrition” AND “Education”) OR (“Nutrition” AND “Instruction”) AND #2 “Control Groups” OR “Research Design” OR “Quasiexperimental Design” OR “Quasi experimental Design”

^a^Search results were limited to English and publication from January 2005 to December 2015

To be included in the systematic review, the articles had to report primary research findings of controlled nutrition education intervention trials from peer-reviewed journals. Included studies could address content other than nutrition, but nutrition had to be a key component. Additionally, included interventions had to focus on health promotion and disease prevention and have an education component. Inclusion criteria also required that interventions consist of more than one session and be conducted in the United States with free-living consumer populations. All included articles were published in English between January 2005 and December 2015. In cases where more than one article from the same study was located, only primary outcomes paper was included in the review to prevent over-representation of the type of control group used.

Excluded articles were studies reporting pilot, feasibility, cross-sectional, follow-up, or secondary analysis findings and those lacking a control or comparison group. Studies that focused on weight loss or disease management/treatment and those lacking an education component (e.g., those solely manipulating environmental factors) also were excluded. Additionally, all studies targeting professionals (e.g., health care, child care) or individuals recruited due to a pre-existing disease, such as diabetes, eating disorders, and obesity, or hospitalization, were excluded.

### Data management

**Fig. 1:**
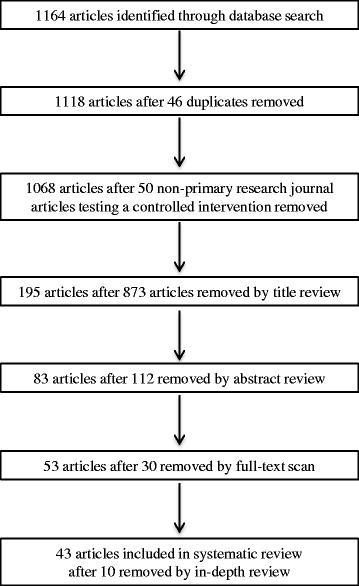
Flow chart of literature search results for controlled research studies reporting (e.g., not secondary analysis or pilot, feasibility, or follow-up studies) results of nutrition education primary-prevention (e.g., not part of treatment for disease or weight loss) interventions consisting of more than one session conducted with free-living individuals in the United States

Citations for the 1164 articles returned by the systematic literature search were entered in a citation management tool (Fig. 1). After removal of duplicates (*n* = 46) and publications that were not complete primary research articles (e.g., commentaries, viewpoints, editorials, letters, survey studies, abstracts, review articles, *n* = 50), two members of the study team independently conducted an initial screening of all article titles to identify those congruent with the study purpose. The title review yielded 195 articles that appeared to meet inclusion criteria. Next, article abstracts were independently reviewed by the same team members and 83 were identified as congruent with study purposes. Four team members scanned the articles and identified 53 articles meeting inclusion criteria. During data extraction, 10 additional articles were eliminated because they did not meet inclusion criteria thereby yielding a total of 43 reviewed articles.

### Data collection and analysis

After scrutinizing guidance from the Nutrition Education Systematic Review Project [29] and Cochrane Collaboration [31, 32] as well as previously published systematic reviews [33–35], data extraction tables were designed by the study team. These tables were iteratively pilot-tested and refined.

Data were extracted by one team member and independently checked for accuracy by two other team members. As shown in Table 3, the factors extracted included treatment provided to the experimental and control groups, overall intervention content, procedures used to tailor the intervention to participants, intervention delivery mode (e.g., group, individual), intervention format (e.g., curriculum, website, brochure) and duration, intervention setting, individual intervention session description (e.g., number of sessions or interactions, session duration, session frequency, content of each session, time allotment for each session component, overall duration of the intervention), procedures for standardizing intervention across multiple sites/practitioners, procedures for assessing fidelity of implementation across multiple sites, and procedures for blinding (masking) participants and/or intervention staff to participant group assignment, rationale for control group type selected, as well as sample size, attrition rate, and theoretical foundation. The goal of the factors extracted was to document the explicit presence or absence of each factor reported in the article. Additionally, only the 43 articles identified in the search were reviewed; extracting additional data from bibliographical references to previous developmental work cited in articles was beyond the scope of this study. A written narrative describing the treatment groups was prepared for each study. Extraction tables were content analyzed by team members to identify themes used to prepare a narrative synthesis of findings.

**Table 3:** Factors Extracted in Systematic Review^a^

Description of…
1. … overall intervention content (i.e., content provided)
2. … how intervention was tailored to participants
3. … how intervention was delivered (e.g., individual or group)
4. … intervention material type used to provide content (e.g., curriculum, website, brochure, etc.)
5. … total duration of intervention: (e.g., 6 weeks)
6. … intervention setting (e.g., WIC office, home)
7. … individual sessions
a. number of individual sessions or # of interactions (e.g., newsletters)
b. duration of individual sessions or length of materials (e.g., #pages)
c. frequency of individual sessions (e.g., 1 session weekly for 6 weeks)
d. content of each session/interaction (e.g., week 1: carbohydrates; week 2: protein, etc.)
e. duration of each main component of each individual session (e.g., 10 min on food safety lecture, 30 min on food preparation activities; 1 page on dairy products, ½ page on fruits)
8. … procedures for standardization of intervention across centers/researcher staff/practitioners/implementers (e.g., personnel guided by manuals, guidelines, standard operating procedures, training; participants used standardized materials such as newsletters, videos, websites, curricula)
9. … procedures for assessing intervention implementation with fidelity to that planned (e.g., staff supervised during implementation or videotaped/observed to ensure implementation was as planned; staff surveys to describe what they did during the intervention)
10. … procedures for blinding participant and researcher to treatment group assignment. If researchers were not blind, procedures for preventing differential treatment.
11. …rationale for selection of control group type.
12. Reference for Instructional Materials Used
13. Theoretical underpinnings of intervention

^a^Modeled closely from Boutron et al. [94] and Tolsgaard et al. [95]

## Results

The treatment provided to the experimental and control conditions in the studies meeting the inclusion criteria are described in Table 4. For accuracy, these descriptions used verbiage from the original research inasmuch as possible [36]. More than one-third of the 43 studies in the review had an inactive control condition; that is, the control group received no treatment or delayed-treatment (or wait-list). Because a key goal of this study was to compare how control and experimental conditions are described in peer-reviewed literature, results will focus on the 28 studies that had an active control condition. Of these studies, 7 had a usual or standard treatment for the control group, 12 offered an alternative active treatment to control participants, and 9 were dismantling (or additive) component active controls (2 of the 9 were mixed in that control groups received an alternative active treatment whereas the experimental groups received additive treatments).

**Table 4:** Description of experimental and control group treatments of nutrition education interventions (*n* = 43)

Author, Date	Experimental Group	Control (Comparison) Group
Powers et al., 2005 [71]	*N* = 702 at posttest Attrition: Not reported Treatment: Over 6 weeks, 2nd and 3rd graders in low-income school district received 6 group classes, delivered by educators using a curricular guide and materials, on nutrition, including diary intake, fruit and vegetable (F/V) intake, Food Guide Pyramid knowledge, nutrient-food association knowledge, and nutrient-job association knowledge. Concepts enhanced with hand-on activities, bulletin boards, role modeling by nutrition educators.	*N* = 398 at posttest Attrition: Not reported Treatment: None
Kemirembe et al., 2011 [72]	*N* = 43 at posttest Attrition: Not reported Treatment: Low-income youth participating in the Expanded Food and Nutrition Education Program received 4 2- to 3-h hands-on/experiential group sessions based on 5 lessons from *Up for the Challenge: Health, Fitness, and Nutrition* curriculum over 4 weeks. Sessions focused on nutrition knowledge, skills, making healthy food choices.	*N* = 43 at posttest Attrition: Not reported Treatment: None reported
Katz et al., 2011 [70]	*N* = 628 at baseline Attrition: Not reported Treatment: 2nd to 4th grade children received 4 20-min *Nutrition Detectives* group sessions taught by physical education teachers using Powerpoint presentations and demonstrations, and hand-on activities about selecting healthy foods (minimally processed, nutrient dense, low in added sugars and trans-fat, and rich in desirable constituents, such as fiber) and a booster session 3 months later.	*N* = 552 at baseline Attrition: Not reported Treatment: None
Keihner et al., 2011 [96]	*N* = 703 at baseline; varied with measure at posttest highest *n* = 698 Attrition: 11% Treatment: 4th and 5th grade children received 10 50-min group *Power Play* sessions on nutrition and physical activity taught by trained teachers over 8 weeks using lesson plans, student workbooks, cookbooks, parent brochures, songs, posters, and stickers. Teachers completed tracking forms to document implementation.	*N* = 451 at baseline; varied with measure at posttest highest *n* = 448 Attrition:14% Treatment: None
Backman et al., 2011 [67]	*N* = 186 at baseline; 156 at post-test Attrition:16% Treatment: Low-income African American women received 6 1-h group sessions, tailored to their culture, selected from the *Toolbox of Tailored Educational Lessons to Improve F/v and Physical Activity Behaviors,* handouts, and resource materials weekly for 6 weeks, taught by trained health educators; sessions included presentations, discussion, and problem solving; staff attended all sessions to ensure fidelity and quality of session delivery.	*N* = 199 at baseline; 171 post-test Attrition:14% Treatment: None
Roofe et al., 2011 [97]	*N* = 79 Attrition: Not reported Treatment: Kindergarteners received 30-min nutrition lessons over a period of 1 month on My Pyramid, calcium, F/V, and nutritional health by trained college students; lesson consisted of a story, game, and coloring sheet to take home; printed parent education materials were provided.	*N* = 77 Attrition: Not reported Treatment: None
McCarthy et al., 2012 [98]	*N* = 613 completed at least pre or posttest; 454 completed both pre and posttest^a^ Attrition: Not reported Treatment: Low-income middle school children received face-to-face instruction using the Harvest of the Month exposure-based nutrition education intervention that promotes F/V intake with monthly in-class F/V tasting activities, informational materials provided to teachers, parent newsletters, promotional posters and banners, related books in the school library, informative pages in the students’ day planners, and school bulletin announcements; program lasted 7 months.	*N* = 396 completed at least pre or posttest; 276 completed both pre and post-test^a^ Attrition: Not reported Treatment: None
Alaimo et al., 2015 [69]	Year 1: *N* = 320 baseline, 281 posttest. Year 2: 367 baseline, 281 posttest Attrition: 12% (year 1), 23% (year 2) Treatment: 3rd, 4th, and 5th grade teachers were trained and encouraged to offer 20 h of classroom-based nutrition education per year to their students; teachers were given nutrition education resources/support including newsletters and classroom nutrition education kits, healthy eating coaching in the cafeteria, and taste testing; teachers were encouraged to sign up for the YMCA “Nutrition in Action” program (a 6 week nutrition education program taught in the classroom by YMCA representatives), provided with non-food reward boxes, and social marketing materials (e.g., Project FIT health messages through mini-media, branded promotional materials, and wellness event ideas); the program also provided wellness training for after-school staff; improvements in school policies, programs, and environment though Health School Action Tools with a trained facilitator; and parent nutrition education.	Year 1: *N* = 114 baseline, 105 posttest. Year 2: 104 baseline, 96 posttest. Attrition: 8% (year 1), 8% (year 2) Treatment: None
Townsend et al., 2008 [22]	*N* = 162 youth groups of 3586 participants completed pre/post Attrition: not reported Treatment: Low-income 9–11 year-old children participating in EFNEP received 7 *Eating Right Is Basic for 9–11 years olds* 1-h group lessons in ~6 to 8 weeks to increase awareness of F/V in healthful diets and food safety; lessons taught by trained staff. Curriculum aimed to enhance knowledge, skills, and food choices using experiential activities (e.g., food tasting, food art, food puzzles and games, and preparation of F/V).	*N* = 67 youth groups of 1526 completed pre/post Attrition: not reported Treatment: Experimental group treatment provided after the post-test.
Eicher-Miller, et al., 2009 [99]	N=137^a^ Attrition:7%^a^ Treatment: Over 5 weeks, female participants in the Food Stamp Nutrition Education (FSNE) program received 1 30–60 min group session on MyPyramid before baseline measurement then 4 weekly 30–60 min interactive sessions delivered by trained FSNE personnel on food groups, food safety, food shopping/resource management, and wellness and included demonstrations, discussions, hands-on activities, and other active learning strategies. Food preparation modeling,.Lessons for the sessions were tailored to the participant age and household composition and were taught in private homes or community centers.	N=82^a^ Attrition: 7%^a^ Treatment: Food stamp nutrition education participants received 1 30–60 min session before baseline measurement and received the additional 4 sessions after the post-test (delayed treatment).
Wilcox et al., 2013 [68]	Baseline: *N* = 38 churches, *n* = 749; Posttest: 37 churches, *n* = 466 Attrition:38% Treatment: African-American church members received Faith, Activity, and Nutrition (FAN) activities focused on physical activity and healthy eating availability and accessibility; physical structures; social structures; cultural and media messages. Churches had flexibility in activities offered, but all included church bulletin inserts, messages from the pulpit, educational materials, project bulletin board, and physical activity and healthy eating policy/practices the pastor could set. Program implemented by committee of 5 church members who competed 8-h training and developed a formal action plan supporting physical activity and dietary change; 2 were trained on the Dietary Approaches to Stop Hypertension (DASH) diet plan. The committee received monthly mailing for 15 months about strategies for changing physical activity or healthy eating behaviors, program messages, handouts to give to church members, tools for cooks, and follow-up technical assistance calls.	Baseline 36 churches, *n* = 508; posttest 33 churches, *n* = 307 Attrition:40% Treatment: Delayed treatment
Bogart et al., 2014 [73]	*N* = 1515 baseline, 2997 posttest^a^ Attrition: 7%^a^ Treatment: 7th grade students enrolled in a school with environmental changes (e.g., greater F/V variety at lunch, free chilled filtered water at lunch, cafeteria point-of-sale signage and posters). Trained peer leader club members offered 2 lunchtime social marketing sessions/week for 5 weeks discussing SNaX messages; film, poster handouts, bookmarks, parent-student activities.	*N* = 1524 baseline, 2997 posttest^a^ Attrition:7%^a^ Treatment: Delayed treatment
Dollahite et al., 2014 [62]	*N* = 85 baseline; 74 posttest Attrition:13% Treatment: Low-income parents received 8 weekly nutrition education group sessions using the Eating Right is Basic-Enhanced curriculum, facilitated by 8 trained paraprofessionals aiming to increase knowledge, skills, food choices, and goal setting using hands on activities, discussion, and food preparation and tasting. Topics included portion sizes, MyPyramid food groups, food safety, food shopping, menu planning and feeding children.	*N* = 83 baseline; 60 posttest Attrition:28% Treatment: Delayed treatment
Kattelmann et al., 2014 [66]	*N* = 824 baseline; *n* = 618 posttest; *n* = 497 followup Attrition: 25% (baseline to posttest); 40% (baseline to follow-up) Treatment: Over a 10-week period, college students had access to 21 mini-web-based lessons to foster healthy weight-related lifestyle behaviors (eating behavior, physical activity, stress management, and non-diet approach to weight management; viewing lessons was not required) and received 3 weekly email nudges (short, entertaining, stage-tailored messages with videos personalized to participant stage of change for F/V consumption, physical activity, and stress management) and 1 nudge reminding them view new lessons, and set goals each week for 1 to 3 targeted behaviors.	*N* = 815 baseline; *n* = 623 posttest, *n* = 476 follow-up Attrition: 24% (baseline to posttest); 42% (baseline to follow-up) Treatment: Delayed treatment
Madsen et al., 2015 [100]	*N* = 583 baseline Attrition: 24%^a^ Treatment: Children in 3rd-5th grade enrolled in low-income school districts were taught for 12-weeks by a registered dietitian using the EB4 K with Play, a multicomponent school-based nutrition and energy balance intervention that included food tastings, physical activity games, strategies to help students meet physical activity and nutrition goals; a registered dietitian worked with school staff and parents to implement wellness policies and improvements in school food service; a play coach offered structured active recess activities before and during school and led a physical activity sessions every other week and 4 afterschool 5-week long sports leagues throughout the year. Teachers were trained to implement Play works games and management strategies in students’ physical education sessions.	*N* = 296 baseline Attrition: 24%^a^ Treatment: Delayed treatment
Hopper et al., 2005 [47]	*N* = 142 Attrition: Not reported Treatment: For 20-weeks (10 in fall and 10 in spring), trained teachers taught 3rd grade school students 3 30-min physical education group sessions per week that emphasized fitness, tips on walking and biking with parents, and included activities and games and 20 min of aerobic activity in each; 2 30-min nutrition group sessions per week emphasizing nutrition and heart health, reading labels, and tips on how to discuss healthy eating with parents, and included hands-on activities, games, group discussion, and role-playing; and each week a packet of exercise and nutrition activities was sent home for parents and children to use together and then returned the following week. Parents attended orientation session, received feedback on their own and their children’s health measures (e.g., height, weight, blood cholesterol, dietary intake), and asked to set activity goals, and taught to log family fitness activities; teachers taught children to log family fitness activities in cases where their parents did not attend the orientation. Parents and children received points for meeting weekly goals, stickers, and t-shirts. Teachers received 10-h training and ongoing assistance from researchers.	*N* = 96 Attrition: not reported Treatment: Elementary school students received nothing beyond the traditional physical education and nutrition education program at school. Experimental curriculum and parent materials supplied after posttest.
Pobocik et al., 2009 [41]	*N* = 45 Attrition: 8% ^a^ Treatment: 8th grade students enrolled in 2 Family and Consumer Sciences classes combined to receive 5 *Do Dairy* group sessions on 5 consecutive days with the main topics of: energy balance, health benefits of dairy, serving sizes, overcoming barriers to dairy intake, bone health, meal planning, goal setting; 20 min of each session was information presentation and 25 min was testing, activities, and demonstrations. Classes conducted by dietetic interns.	*N* = 18 Attrition: 8% ^a^ Treatment: 7th grade students enrolled in 1 Family and Consumer Sciences class received regular instruction.
Dzewaltowski et al., 2010 [55]	*N* = 148 baseline; 134 posttest Attrition:9% Treatment: 4th grade school children participated in an after school program delivered by staff who had been trained by Cooperative Extension personnel via 3 trainings/year for 2 years, monthly meetings, continuous web support. Trained staff provided children with 30-min/day of organized physical activity based on CATCH Kids Club Physical Activity, coordinated with school food service to provide F/V daily snack, and 60-min/week group sessions on physical activity, F/V, reduced TV/video game time, and removing TV from bedroom that were presented in 15 sessions in fall and 14 sessions in spring each year for 2 years. Incorporated activities related to modifying home environments. Researchers visited sites on random days to observe children’s physical activity and fitness instruction and log type of session offered.	*N* = 125 baseline; 112 posttest Attrition:10% Treatment: 4th grade school children participated in after school program. Researchers visited sites on random days to observe children’s physical activity and fitness instruction and log type of session offered.
Bensley et al., 2011 [37]	*N* = 243 Attrition: 52%^a^ Treatment: WIC participants received 2 F/V nutrition education modules based on wichealth.org via internet; education based on participant stages of change, interests, and needs. Participants selected whether to receive education by internet or in traditional group session. Participants were given option to participate in follow-up motivational negotiation nutrition counseling with trained WIC staff.	*N* = 536 Attrition: 52%^a^ Treatment: WIC participants received traditional F/V nutrition education in the form of group classes at the WIC clinic or a self-guided nutrition education information “mall” (educational material displayed on a bulletin board). Participants were given option to participate in follow-up motivational negotiation nutrition counseling with trained WIC staff.
McCaughtry et al., 2011 [43]	*N* = 1476 Attrition: 8%^a^ Treatment: Middle school health education teacher received 8 h of inservice on constructivist-oriented nutrition education curriculum and implemented it in 6 active learning (e.g., reflection, role playing, discussion, presentations, advertising campaign, home eating analysis, parent interviews) 1-h group sessions over 6 weeks. Content focused on benefits of food groups, eating based on food groups, analyzing influences on eating, food labels, health claims on labels, body image, and fast food. Teachers kept detailed teaching logs and were observed at random to ensure curriculum was implemented with fidelity.	*N* = 656 Attrition:8%^a^ Treatment: Middle school children received health education with no nutrition content during the study period. Teachers kept detailed teaching logs and were observed at random to ensure no nutrition was taught during study period.
Wall et al., 2012 [40]	*N* = 1187 baseline, 1047 post-test Attrition:12% Treatment: Over a 3- to 5-week period, children enrolled in SNAP-Ed participating elementary school received 4 vegetable education group sessions from instructors who were trained via a webinar.	*N* = 1044 baseline; 890 post-test Attrition:15% Treatment: Children enrolled in SNAP-Ed participating elementary schools did not receive vegetable related instruction but other nutrition instruction (e.g., whole grains) or physical activity was not prohibited.
Herbert et al., 2013 [38]	*N* = 59 Attrition: 0% Treatment: For 12 weeks, 3rd and 4th grade students received 1 weekly 60-min group session from the Energize curriculum facilitated by 2–3 Energize instructors (i.e., nongovernmental organization staff that specialize in exercise training or recreation therapy, AmeriCorps volunteers and college interns). Each session was 15 min of nutrition education (different topics for each week), 10-min warm-up, and 35 min of aerobic exercise activities and fitness games. Instructors met weekly with the Energy director weekly. Intervention content focused on food pyramid, grains, and F/V.	*N* = 45 Attrition: 0% Treatment: 3rd and 4th grade students participated in normal classroom activities.
Devine et al., 2005 [45]	*N* = 201 completed both pretest and post-test Attrition: 32% Treatment: Low-income women participating in community programs received 6 90-min weekly small group sessions facilitated by trained community nutrition paraprofessionals; 1 session focused on participants’ familiarity with and preferences for F/V; 1 session focused on F/V recommendations, intake, and portions; remaining 4 sessions were selected by participants from these topics: salads, soup, smart grocery shopping, quick meals, kids and vegetables, eating out, and fruit. Each session included a warm-up activity, food preparation and tasting experience, group-learning activity, take-home activity, and opportunity to give feedback on that session and plan for the next one.	*N* = 68 completed both pretest and post-test Attrition: 34% Treatment: Low-income women participating in community programs received *Eat 5 Fruits and Vegetables Every Day* pamphlet. They were participants in a 6 week parenting or budgeting community education program.
Nitzke et al., 2006 [48]	*N* = 2024 at baseline; 1255 at posttest ^a^ Attrition: 38% Treatment: Economically disadvantaged college students were mailed colorful, stage of change-tailored newsletters on F/V and a related magazine monthly for 8 months; received mailed computer-generated reports tailored to participant F/V intake and Transtheoretical Model stage of change and other constructs after baseline and study mid-point assessment; and received educational phone calls from trained staff using a protocol 4 weeks after the initial and mid-point assessment	*N* = 2024 at baseline; 1255 at posttest ^a^ Attrition: 38% Treatment: College students received mailed, non-tailored, publically available pamphlet on F/V.
Mitchell et al., 2006 [52]	*N* = 425 baseline, 280 post-test Attrition: 34% Treatment: Limited resource older adults in congregate nutrition sites received 5 *Pills, Potions, and Powders* group sessions over 9 weeks focused on the appropriate use and potential consequences of herbal and other dietary supplements, and importance of reviewing use with one’s health care professionals. Sessions 1 to 3: practical information related to herbal products and dietary supplements in general. Session 4: micronutrients of particular concern to older adults (i.e., vitamin D, vitamin B-12, calcium). Session 5: participants developed personal action plan to carry out intervention-related strategies. Instructional activities were designed to influence self-efficacy and outcome expectations. Classes conducted by trained Family & Consumer Science County Agents. Instructors reported deviations from curriculum protocol.	*N* = 581 baseline; 423 post-test Attrition: 27% Treatment: Limited resource older adults in congregate nutrition sites received 5 *Weighty Matters* group sessions focused on weight management and exercise. Sessions 1 to 4: weight management and exercise. Session 5: participants developed a personal action plan to carry out intervention-related strategies. Classes were conducted by trained Family & Consumer Science County Agents. Instructors reported deviations from curriculum protocol.
McCarthy et al., 2007 [51]	*N* = 188 at baseline 101 retained for analysis Attrition: not reported Treatment: Healthy African-American women received 8 weekly 90 min *Fight Cancer with Fitness* group sessions focused on skills training in a balanced regular exercise regimen (muscle strengthening, flexibility enhancement, and aerobic conditioning); low-fat, complex carbohydrate-rich (high-fiber) diet; and cancer-preventive benefits of increased quantity and variety of F/V intake. Dietary assessment and feedback from a dietitian 3 times during the intervention was provided. Participants were encouraged to invite one close female relative or friend to provide social support post-intervention.	*N* = 178 at baseline; 87 retained for analysis Attrition: not reported Treatment: Healthy African-American women received 8 weekly 90-min group sessions focused on current African-American women’s topics; group sessions addressed cancer-related topics (i.e., barriers to and facilitators of tobacco control, screening behaviors for breast, cervical, uterine, colorectal, prostate, and skin cancer;) and non-cancer topics (i.e., menopause, depression). Guest role models attended and videos were used. There was no exercise or external social support component. Control group instructors were not involved in the experimental group treatment.
Cook et al., 2007 [49]	*N* = 236 baseline received intervention; 209 post-test Attrition:11% Treatment: For 3 months, employees at a human resources provider had access to *Health Connection*, a comprehensive multimedia, highly interactive web-based health promotion program that offered information and guidance on stress management, nutrition/weight management, and fitness/physical activity and provided opportunities for observational learning, building self-efficacy and self-tailoring of content and sequence. Screen shots and outline of web program content provided.	*N* = 230 baseline received intervention; 210 post-test Attrition:9% Treatment: Employees at a human resources provider received a packet of 5 printed commercially available booklets covering the same topics as the web-based program (not necessarily the same content) that included tracking forms and logs. Booklet outlines provided.
Greene et al., 2008 [56]	*N* = 1277^a^; 410 with complete data Attrition: 35% ^a^ calculated from paper Treatment: Older adults received a manual on F/V intake organized by stages and processes of change, and included recipes and tips for increasing F/V intake; 3 4-month cycles of receiving a monthly stage of change-based F/V newsletter promoting self-efficacy and decisional balance for 3 months and a tailored report providing personalized feedback for 1 month. They also received 15-min coaching calls by trained counselors for follow-up 4–6 weeks after each personalized feedback report was sent. Personalized feedback reports were based on interviews at baseline and months 4 and 8.	*N* = 1277^a^; 424 with complete data Attrition: 35%^a^ calculated from paper Treatment: Older adults received a manual on exercise or fall-prevention, neither included information on nutrition.
Wolf et al., 2009 [57]	*N* = 246 baseline; 216 post-test Attrition:12% Treatment: Urban and mostly immigrant black men mailed “*Men Eat 9 A Day*” brochure, received a maximum of 2 tailored telephone education (TTE) calls within 1 month period (within 2 weeks of randomization); initial call averaged 20 min. TTE aimed to increase F/V intake and raise awareness about importance of eating a variety of F/V, recommended intakes, appropriate portion sizes, and potential health benefits, overcome barriers, provide support, set goals. A brief (average 5 min) follow-up call was made if necessary. Treatment fidelity checks were conducted on recordings of calls.	*N* = 244 baseline; 215 post-test Attrition:12% Treatment: Urban and mostly immigrant black men were mailed brochure on prostate cancer and received a maximum of 2 TTE calls within 1 month period (within 2 weeks of randomization. The initial call provided on prostate cancer education (average 20 min long). A brief follow-up call was made if necessary (average 5 min).
Clifford et al., 2009 [50]	*N* = 50 baseline & post-test; 30 completed 4 month follow-up Attrition: 0% post-test; 11% at follow-up Treatment: College students viewed *Good Grubbin’*, 4 15-min web-based videos on cooking, nutrition, and F/V viewed over a 4-week period. Subtopics for each episode were: weight loss, cooking vegetarian, grilling for a group, and storing F/V. Videos featured a student struggling with a meal-planning or nutrition issue, discussion of the issue with friends, working with a dietitian to identify strategies for coping with the issue, and concluded with an interview about how the video helped the participant to successfully address the issue. Videos were set primarily in a kitchen and supermarket. Students completed a short form after viewing each episode to track compliance with treatment.	*N* = 51 at baseline & posttest; 30 completed 4 month follow-up Attrition: 0% at post-test; 11% at follow-up Treatment: College students viewed 4 5-min web-based videos on sleep disorders over 4 weeks. Students completed a short form after viewing each episode to track compliance with treatment.
Hekler et al., 2010 [44]	*N* = 28 calculated from paper Attrition: 10% Treatment: Undergraduate students enrolled in a food-related social issues course taught by study authors read selected portions of popular books (e.g., Michael Pollan’s *Omnivore’s Dilemma*); watched documentaries (e.g., Morgan Spurlock’s *Supersize Me*); discussed major themes of food-related social issues in class; wrote a newspaper opinion article; and created a video in small groups advocating behavior change related to a course theme. Students were encouraged to find food-related social issue organizations, attend events, and share experiences.	*N* = 72 calculated from paper Attrition: 14% Treatment: Undergraduate students enrolled in 1 of 3 upper-level health- or obesity-related courses (Health Psychology, Community Assessment/Health, Obesity:Clinical/Societal Implications) taught by experienced health promotion researchers.
Glanz et al., 2012 [60]	*N* = 128 (completed all assessments) Attrition:11%^a^ Treatment: Adult primary household food shopper/preparers with children who were interested in improving their diets received an 8-week nutrient-rich foods (NRF) education program consisting of 1-h face-to-face educational session led by a registered dietitian consisting of a 15-min video, hands-on exercise, and review of program tools; access to a website to look up NRF food scores; toll-free telephone number to reach a dietitian; weekly motivational and reminder email messages; and biweekly mailings.	*N* = 61 (completed all assessments) Attrition:11%^a^ Treatment: Adult primary household food shopper/preparers with children who were interested in improving their diets received an 8-week program comprised of a 1-h education session led by a registered dietitian that emphasized general nutrition guidance, consisting of a 15-min video about the Dietary Guidelines for Americans and MyPyramid; an information sheet and group exercise on the Nutrition Facts Panel; and 2 mailings of government produced nutrition brochures.
McClelland et al., 2013 [42]	N=463^a^; at least 172 at follow-up Attrition: 30%^a^ Limited-resource, older adults received 5 weekly group sessions from “Eat Smart, Stay Well”; topics included healthy diet, effects of dietary fats, benefits of F/V, and strategies for making healthy choices. All sessions were taught by County Extension Agents and included progress check-ups, discussions, food preparation demonstrations, interactive hands-on skill-building activities, taste tests, challenges, and peer-group exchange. At the end of 5 weeks, participants received the control group treatment for the 5 weeks (crossover design).	N=463^a^; at least 152 at follow-up Attrition: 30%^a^ Treatment: Elderly adults received 5 weekly group sessions from the “Eating Well on a Budget” curriculum which focused on food dollar management to increase nutritious foods purchased within a limited budget. All sessions were taught by County Extension Agents and included weekly progress check-ups, discussions, food preparation demonstrations, interactive hands-on skill-building activities, taste tests, challenges, and peer-group exchange. At the end of 5 weeks, participants received the experimental group treatment.
Healy et al., 2015 [39]	*N* = 22 Attrition: Not reported Treatment: High school students received 50-min daily in 7 sessions held over 1.5 weeks on the 10 principles of intuitive eating and guidelines to follow each principle as part of a health and physical education class. Instruction format included lecture, discussion, question/answer, and group activities.	*N* = 26 Attrition: Not reported Treatment: High school students received 50-min daily in 7 sessions held over 1.5 weeks on “Destination Wellness” that teaches how to distinguish between science and hype when searching for nutrition information, define a realistic and healthy body image, understand historical trends in body images in the media, and healthy eating as part of a health and physical education class. Lesson format included lecture, discussion, question/answer, and group activities.
Elder et al., 2009 [64]	Experimental Group 1 *N* = 120 Attrition: Not reported Treatment: Each week for 12 weeks, Spanish-speaking Mexican/Mexican-American women received a tailored newsletter and homework assignments by mail. Newsletter content was tailored based on focus group data, participant observations, baseline data, and stage of change and included tips on reducing fiber and fat and increasing F/V, overcoming barriers to F/V and lowfat food consumption, outcome expectations for a healthy diet, and family support and interaction for a healthy diet. The first and last newsletter provided feedback on health behaviors and promoted goal setting. Participants received weekly home visits or phone visits from extensively project-trained bicultural/bilingual promotoras (lay health advisors/counselors) to review the newsletter and complete homework assignment together if the participant requested assistance. Experimental Group 2 *N* = 118 Attrition: Not reported Treatment: Participants were mailed the same newsletters and similar homework assignment as Experimental Group 1 but did not receive promotora visits.	*N* = 119 Attrition: Not reported Treatment: Each week for 12 weeks, Spanish-speaking Mexican/Mexican-American women were mailed newsletters produced by other organization that were on similar topics, but not tailored.
Resnicow et al., 2009 [53]	*N* = 372 baseline; 304 at 3 month follow-up Attrition: 18% Treatment: African-American adults were mailed 3 tailored 8- to 12-page newsletters focusing on increasing F/V intake 1/month for 3 months. Newsletters included 2 recipe cards with small bags of spices and either a notepad or magnet with F/V serving sizes. Newsletters were personalized with participant’s name and tailored to F/V intake, demographics, and 1 of 16 ethnic identities using messages and graphic images.	*N* = 188 at baseline; 164 at 3 month follow-up Attrition: 13% Treatment: African-America adults received same treatment as experimental group except newsletters tailoring did not include ethnic identity, but were designed for a general black American audience and used ethnically neutral images.
Gans et al., 2009 [58]	Experimental Group 1 *N* = 454 Attrition: 42%^a^ Treatment: Low-income adults were mailed 1 packet of nutrition education materials from Your Healthy Life/Su Vida Saludable tailored to low income, ethnically diverse adults. Topics included increasing F/V and reducing fat. Materials mailed with 3-ring binder, magnet shopping list, 10 min motivational/instructional video. Experimental Group 2 *N* = 474 Attrition: 42%^a^ Treatment: Over 12 weeks, low-income adults were mailed nutrition education materials similar to the materials sent to Experimental Group 1 excepted they were divided up and send in 4 separate mailings. Experimental Group 3 *N* = 462 Attrition: 42%^a^ Treatment: Over 12 weeks, low-income adults were mailed nutrition education materials similar to the materials sent to Experimental Group 2; the first packet was similar to Experimental Group 2, remaining packets were re-tailored based on feedback collected via brief telephone surveys 2 weeks before mailing packets 2 to 4.	*N* = 451 Attrition: 42%^a^ Treatment: Low-income adults were mailed 1 packet of non-tailored nutrition brochures from national health promotion agencies that contained ~60 pages of nutrition information related to lowering fat and increasing F/V intake.
Alexander et al., 2010 [63]	Experimental Group 1 *N* = 839 baseline; 613 at follow-up (based on Table 2) Attrition:27% Treatment: Adult health plan members received 4 F/V online sessions 1, 3, 13, and 15 weeks after enrollment. Automated emails notified participants that new web session was available. Each session had 4–5 pages of core content, illustrations, optional links, and supplemental information (e.g., illustrations, videos, audio files,) tailored to participant stage of change for F/V intake, motivation to change, barriers, and cues to action; optional tailored menus offered; 60-s recipe preparation videos were available. Experimental Group 2 *N* = 838 baseline; 588 at follow-up Attrition:30% Treatment: Received same treatment as Experimental Group 1 plus up to 4 e-mailed motivational interviewing counseling sessions which were initiated within 1 week following each new web session visit and were conducted by trained assistants.	*N* = 836 baseline, 619 at follow-up Attrition:26% Treatment: Same treatment as Experimental Group 1 except were given general F/V information without tailoring.
Hughes et al., 2011 [59]	Experimental Group 1 *N* = 150 baseline; 128 6-month follow-up, 137 12-month follow-up Attrition:15% at 6 months; 9% at 12 months Older workers completed an in-person baseline interview with a health professional. After group assignment, participants met with a trained staff member who conducted in-person health-risk assessments, discussion about health behavior changes participant wanted to adopt, and negotiated an action plan for meeting change goals. In the following week, participants were contacted by phone to assess success; those having difficulty had a second meeting, thereafter participants were contacted by email or phone biweekly for 6 months and monthly for the next 6 months. Contacts focused on plan reevaluation and setting other goals. In-person assessments were conducted after 6 and 12 months, and more frequently if needed. Experimental Group 2 *N* = 135 baseline; 110 6-month follow-up, 114 12-month follow-up Attrition:19% at 6 months; 16% at 12 months After completing baseline interview, older workers received an email message to visit the project website which contained a survey that gathered information used to generate an individual risk profile, identify areas participants could work on to improve health, help participants create plans to meet behavior goals, and track participant visits. Website documented participant visits.	*N* = 138 baseline; 122 6-month follow-up, 116 12-month follow-up Attrition:12% at 6 months; 16% at 12 months Treatment: At the baseline interview, older workers were given printed health-promotion materials listing local health-promotion programs and services.
Ratcliffe et al., 2011 [61]	*N* = 170 pretest; 137 post-test Attrition: 19% Treatment: Middle school science class students received regular health and science instruction plus 1-h/week garden-based group sessions for 13 weeks; for each session, 20 min were in the classroom or garden focusing on curricular and gardening activities, 40 min were hands-on gardening experiences, including planting, tending, harvesting, preparing and eating. Community events included a “salad day” for students to serve peers lettuce they had grown and a Saturday “garden party” for friends and family.	*N* = 150 pretest; 99 post-test Attrition: 34% Treatment: Middle school students covered same health and science learning objectives, but no gardening-related activities.
Gans et al., 2015 [54]	Experimental Group 1 *N* = 897 Attrition:16%^a^ (no difference between groups) Treatment: Worksite employees mailed 3 sets of tailored written materials (mailed 1 week after baseline measures, 4 weeks after first mailing, and 4 weeks after second mailing) focusing on increasing F/V and decreasing fat intake; tailoring based on baseline data (F/V and fat intake, participant interest) and 2 other brief “re-tailoring” assessments (1 after second and third mailing). Participants received 28 tailored topics split over the 3 mailings out of 56 possible topics. Experimental Group 2 *N* = 811 Attrition:16%^a^ (no difference between groups) Treatment: Worksite employees received same materials as Experimental Group 1 as well as 3 1-h tailored nutrition-related videotapes with 24 segments of 46 possible.	*N* = 817 Attrition:16%^a^ (no difference between groups) Treatment: Worksite employees mailed 3 sets of traditional (non-tailored), nationally available nutrition education and wellness brochures with content similar to that of the Experimental Groups. They could get tailored materials at end of study.
Franko et al., 2008 [65]	Experimental Group 1 *N* = 165 baseline; 155 post-test; 145 3-month follow-up; 139 6-month follow-up Attrition:16% (baseline to 6-month follow-up) Treatment: College students in Experimental Group 1 received 2 45-min interactive web sessions over 3 weeks of *MyStudentBody.com* *-Nutrition,* 3 information links, 4 main topic pages (“Nutrition 101, Eating on the Run;” “Weighing In” and “Fitness”), self-assessments, and resources. Participants given instructions indicating all areas of website to visit and completed checklist verifying they visited the areas. Experimental Group 2 *N* = 164 baseline; 153 post-test; 139 3-month follow-up; 148 6-month follow-up Attrition:10% (baseline to 6-month follow-up) Treatment: Experimental Group 2 received the same treatment as Experimental Group 1 plus 45-min booster session delivered 3 weeks after the post-test via website when participants were able to choose areas of the website to review.	*N* = 147 baseline; 136 post-test; 136 3-month follow-up; 135 6-month follow-up Attrition:8% (baseline to 6-month follow-up) Treatment: College students were instructed to use an interactive anatomy education website for 2 45-min web sessions.
Ievers-Landis et al., 2005 [46]	Experimental Group 1 *N* = 73 Attrition: 37%^a^ Treatment: Girl Scouts 8 to 11 years-old received 6 30-min group sessions over 6–20 weeks on osteoporosis prevention: osteoporosis overview & healthy eating, osteoporosis prevention & healthy food choices, weight-bearing physical activity & supports for physical activity, barriers and problem-solving techniques, prepared instructional materials to use in peer instruction, prepared high-calcium snacks. Sessions were taught by trained assistants. Experimental Group 2 *N* = 94 Attrition: 37%^a^ Treatment: Girl Scouts 8 to 11 years-old received the same treatment Experimental Group 1 over 9–22 weeks. Their mothers (or primary caregivers) received 2 group sessions: session 1 coincided with the girls’ session 2 and addressed osteoporosis prevention and how to be effective role models and coaches; mothers’ session 2 coincided with girls’ session 4 and reviewed progress on being role models and coaches; mothers and daughters met together and were given problem-solving training and instruction in a reward system as part of the mother’s second session. Mothers’ sessions were taught by a licensed clinical psychologist.	*N* = 80 Attrition: 37%^a^ Treatment: Girl Scouts 8 to 11 years-old received a healthy-lifestyles educational curriculum consisting of 6 30-min sessions delivered over 6–33 weeks focusing on the food guide pyramid, heart-healthy behaviors, avoiding negative health habits, healthy eating and lifestyles activities and games, preparation of a healthy snack.

^a^Not reported separately for experimental and control group

### Factors extracted in reviewed articles

Additional file 1 Table S5 compares the presence of factors extracted in the systematic review of articles. Each factor is described below, citing examples of studies demonstrating the factor

### Description of overall intervention content

Reviewed articles commonly included a description of the overall intervention content provided. Content tended to focus on increasing fruit and/or vegetable intake, lowering fat intake, and healthy eating in general. The extensiveness of the overall content description for experimental groups ranged from only naming the general topic area (e.g., fruits and vegetables) [37] to listing topics and content addressed [38, 39] to reporting content and participant activities [40–42] and teaching strategies [43–46].

Descriptions of the overall content for the control conditions tended to provide much less detail compared to experimental conditions. For example, among those employing usual or standard treatment, one study indicated only that “control classrooms did not receive vegetable-related instruction” [40],p.39 whereas another study reported that health education with no nutrition content was given [43], with neither indicating what control group participants received. Other descriptions of the control condition of usual treatment studies were equally vague indicating these participants received “traditional”, “regular”, or “normal” lessons [37, 38, 41, 47]. Descriptions of treatment provided to the control groups in some alternative active treatment studies also were vague (e.g., control received pamphlet on fruits and vegetables [48], “packet of 5 printed commercially available booklets [49],” videos on sleep disorders [50]). However, several alternative active treatment investigations were more informative, including content similar in detail to the experimental group [46, 49, 51–53]. Dismantling studies tended to provide the greatest detail about the control condition largely because most experimental conditions were additive to the base formed by the control.

### Description of how the intervention was tailored

Unless a goal of an investigation was to determine the effects of tailoring, little information on this factor was reported for experimental or control conditions regardless of whether a usual or other active control condition was used. In usual treatment control conditions, only one study mentioned tailoring for the experimental group [37]. A few alternative active treatment control condition studies tailored experimental and control treatments to demographic characteristics (e.g., older adult learners, African American women) [51, 52]. Some investigations tailored treatments for experimental groups by allowing participants to choose topics or materials [45, 49], with one study giving both experimental and control groups the ability to select topics [51]. The aim of most dismantling studies was to assess the effects of tailoring (experimental groups) vs not tailoring (control group); thus, tailoring descriptions for the control group generally were not applicable. On the other hand, the relative importance of the tailoring method to study aims made reasonably complete descriptions of this process requisite to report for experimental groups. Gans et al. reported [54] that tailoring was based on participant’s fat, fruit, and vegetable intake and related behaviors, self-identified needed behavior changes, personal motivators, barriers, and other psychosocial issues associated with healthy eating, needs, and interests. Resnicow et al.’s [53] report is notable in that these authors provided a table describing messages and graphic images used to tailor study newsletters.

### Description of intervention delivery mode, material type used, duration, and setting

Across all types of control conditions, investigators consistently reported the intervention delivery mode, with the most common being group sessions or online. Descriptions for experimental conditions tended to express delivery mode in explicit terms whereas for control conditions, it was often left to the reader to decide on the mode using implicit clues. This was particularly the case when the control group received a “usual” treatment without further clarification [40, 41, 43, 47, 55].

The type of material that provided intervention content directed to participants tended to be printed (e.g., brochures, pamphlets, manuals, newsletters) and online (e.g., websites, videos). Interventions delivered by instructors to groups used mostly curricula and “lessons.” Some of the reviewed articles gave bibliographical references, internet links, or other means for obtaining intervention materials, with sources for instructional materials more commonly given for experimental than control groups [38, 40–43, 47, 55–59]. An examination by control group type found that references for resources used to deliver usual treatment to control groups were not included. Among alternative active treatment studies, the material types used with both experimental and control groups had comparably detailed descriptions [39, 42, 51, 60], with some exceptions where great detail about the materials used by the experimental group was provided while giving only limited descriptions of those intended for the control group [44, 48]. Material type descriptions tended to be more even across dismantling studies.

Total duration of the intervention delivered to the experimental group was explicitly stated in nearly all studies reviewed. For control groups, total duration was less likely to be clearly described and frequently had to be deduced from a review of the study timeline (e.g., when the baseline and post-test was administered) and comparison to statements made about the experimental group. The setting where group sessions were delivered normally was overtly indicated (e.g., school, community center). Interventions directed to individuals who received mailed materials or used websites generally only implied the setting as being home or worksite [49, 50, 56, 57] and did not report where participants generally used intervention materials.

### Description of individual intervention sessions

Across all types of control groups, the number of sessions or interactions (e.g., newsletters) usually was explicitly stated for both treatment groups. The duration of individual sessions or length of materials was more commonly reported for experimental than usual treatment control groups; for other types of control groups, duration was somewhat more consistently reported for both treatment groups [48, 61]. Reporting of frequency of sessions was fairly even across experimental and control groups in all types of control conditions except usual treatment, where this information was rarely included.

Reports of the content of individual sessions/interactions were provided in about half the active control articles reviewed with most descriptions being abbreviated for the experimental group and virtually non-existent for the control group. In a few cases, researchers provided a table or figure listing concepts/topics/objectives addressed in each session/interaction for the experimental group [40, 41, 54, 61, 62]. Only 2 studies provided a table describing the content of both the experimental and control treatments [46, 49]. Descriptions of the duration of each main component of individual sessions/interactions were rare. The exceptions were Ratcliffe et al. [61] who stated “[e]ach hour-long session consisted of approximately 20 min of instruction followed by 40 min of hands-on garden experiences”p.38, Herbert et al. [38] who reported “Energize engages children in 1, 60-minute class once a week … by involving them in 15 minutes of nutrition education, a 10-minute warm-up … and 35 minutes of aerobic exercise activities and fitness games”p.781, and Pobocik et al. [41] who indicated “[a]pproximately 20 minutes of the 45-minute class were allotted to presenting information … remaining time … for testing, activities, and demonstrations”p.22. Comparable descriptions for control groups were not included.

### Procedures for standardization across centers/practitioners

Procedures for standardizing the experimental condition intervention delivery across centers/practitioners took several forms, including training instructors [38, 40, 43, 45, 47, 52, 55] and utilizing pre-established curricula (instructional lessons and protocols) [38, 40, 41, 43, 47, 55] and/or instructional materials (e.g., printed materials, videos, websites) [37, 48–50, 56, 57]. Standardization procedures were similarly addressed across types of interventions for the experimental group. In contrast, little information related to standardization of implementation of control group treatments was provided for usual treatment control conditions. In alternative and dismantling active treatment studies, the procedures for standardizing control group treatment were frequently addressed and mostly took the form of pre-established instructional materials [39, 49, 50, 52–54, 56, 57, 59, 63–65].

### Procedures for assessing fidelity of implementation

Only about half of active control studies addressed fidelity of adherence to procedures, with most of these including information about procedures for both the experimental and control conditions. Methods used to establish fidelity of implementation for both experimental and control groups in active control studies where teachers or instructors delivered the treatment included detailed/scripted presentations [43, 46], frequent meetings with researchers [38, 46, 47], random observation/videotaping of instructors [43, 46, 55], teaching/feedback logs [43, 52], and audiotaping [57]. Methods used in active control group studies in which participants self-directed their engagement with pre-established treatments (e.g., web-based, printed materials) included completing forms documenting usage of treatment materials immediately after use [50, 64, 65], self-report posttest survey items that gauged extent of treatment use [53, 58], and website tracking data [59].

The vast majority of active control studies provided little detail about fidelity procedures. One notable exception was McCaughtry et al. [43], who described fidelity procedures as including “very detailed (nearly scripted) lessons in the curriculum…a research assistant [who] conducted randomized school visits to observe each health education teacher’s instruction to guarantee that the control teachers were not teaching nutrition content and that the intervention teachers were implementing the curriculum with fidelity,”p.279. Another noteworthy example was provided by Wolf et al.: “Treatment fidelity checks were conducted on 200 (41%) of the intervention calls. Trained raters listened to audio recordings of the calls and completed a checklist documenting whether specific points were covered and whether the interventionist spoke at an appropriate pace, responded to questions with clear answers and probed at appropriate times” [57],p.34.

### Procedures for blinding participants and researchers to treatment group assignment

Limited attention was given to the issue of blinding participants or researchers in the reviewed articles. In many cases, it was not clear whether participants were blinded (or aware there was a control vs experimental group), although this is a typical component of informed consent procedures. None of the studies providing the control group with usual treatment addressed participant blinding. Two articles blinded participants to group assignment by explaining that they were getting one of two programs or using alternate names for “control” or “experimental” groups. In specific, McCarthy et al. stated “A portion of the script used by project staff read … This is a cancer prevention study to compare two programs designed to help black women reduce their risk of cancer and improve their appearance. The first program involves 8 weekly 2-h sessions on diet and exercise. The second program involves 8 weekly 2-h sessions on current health topics of interest to black women, such as breast cancer and menopause. Both programs will be conducted by black women physicians and other professionals. We'll decide which group you'll be assigned to randomly, for example, by flipping a coin…” [51], p.247. In McClelland et al.’s crossover design study, these researchers assigned participants “to either the *Apples* Group (n=6) with the treatment curriculum … delivered first or the *Beans* Group (n=7) with the control curriculum … delivered first” [42], p.2. Another study reported that participant blinding efforts may not have worked. These researchers stated that “[g]irls, mothers, and troop leaders were masked to their group membership assignment;” but went on to say “because the project was called the Osteoporosis Prevention Project, some individuals in the control troops may have determined their status owing to the generic health focus of the sessions” [46],p.158.

The issue of blinding research staff likely is less important when interventions are automated and participant exposure to staff is minimal or non-existent. However, even when there was significant interaction with staff (e.g., in interventions delivering in-person or phone-based treatments), studies rarely addressed staff blinding. A few investigators reported using different instructors for experimental and control conditions [51, 52], whereas others indicated that instructors were not blind to condition due to the nature of the intervention [46, 55, 57]. Blinding also would have been difficult in some of the dismantling studies where part of the treatment for only one of multiple experimental groups involved live interactions with staff [59, 63]. In a few cases, articles reported that study evaluators were kept blind to participant study group assignment [57, 58, 64].

### Rationale for selection of control group type

Reviewed studies seldom provided a rationale for the type of control group used and for those that did, various reasons were cited. These included convenience and comparability (e.g., “Three comparison [college] courses … were selected because they also were upper-level Human Biology courses, were delivered the same quarter, and were taught by experienced health promotion researchers and focused on a health message” [44], p.544) and relative strength (e.g., “Control group participants received fewer follow-up mailings … [that] resulted in a difference in “attention” between treatment arms, it is nonetheless a stronger design than a no-treatment control group” [60], p.62). Appropriateness to setting and participants also was considered (e.g., “Employees … were … assigned to the Web-based … or the print condition. It was recognized that the print materials could also be effective instruments of health behavior improvement (unlike a no-treatment control group) and could be a challenge as a control group … [and] would be a likely workplace alternative to an online program; therefore, the print group was thought to be an appropriate control group for the study” [49], p.e17). Yet, after finding both interventions yielded similar improvements, the article added to the control group rationale by stating… “[b]ecause it was originally thought that the print materials would form a relatively weak intervention compared to the Web program, a no-treatment control was not included in the design” [49],p.e17. Only 3 studies indicated the rationale for the control group was to control for non-specific effects (i.e.,“[t]he control group provided an intervention of identical intensity and program delivery format as the experimental group, ruling out “attention” effects in the experimental group” [52],p.386, “we used an attention control group to take into account the effect of participation” [65],p.37, and “[t]he purpose of this group was to control for any nonspecific effects from being educated about healthy lifestyles and from contact time and number of sessions … with professionals [46],p.158.”

### Behavior change theory use

Nearly one-quarter of all reviewed studies did not indicate whether a theory was used to guide the intervention. Of those that indicated application of a behavior change theory, more than half used the social cognitive theory and about one-quarter used the transtheoretical model. Most studies named the theory used with little additional explanation of how it was operationalized. The most explicit reporting of theory application was by Pobocik et al. [41], who included a table listing social cognitive theory constructs, definition of the construct, and an example of how the construct was operationalized in the Do Dairy intervention. Of those reporting how theories were applied, several used the stage of change construct for tailoring materials [48, 63, 66] and/or selecting assessment scales [40, 48, 50, 54, 64]. Particularly illustrative of theory use in assessment were the tables Wall et al. [40] and Elder et al. [64] provided that listed theory constructs and corresponding evaluation items.

### Comparison across control condition types

In the 7 investigations using a usual or standard active control condition, consisting of “traditional” or “regular” instruction, participants tended to be children enrolled in school or participants in government sponsored programs—perhaps because these systems have an ongoing program available for comparison. Articles gave fairly complete descriptions of the intervention provided to the experimental group, which were mostly curriculum based. They tended not to indicate if or how interventions were tailored and rarely provided information on the content of each session/interaction or how time was apportioned in each session, although this information may be available in the curricula referenced. With regard to the control group intervention, other than the overall intervention content, delivery (individual or group), and setting, little other information was provided. In most cases, too little information was provided about the usual treatment to determine whether the control group’s treatment was comparable on non-specific factors to that received by the experimental group [38, 40, 41, 47, 55]. Descriptions in one study, which compared differences in teaching strategies (e.g., traditional vs. tailored online) indicated fairly similar attention to non-specific factors [37].

In the 12 studies providing an alternative active treatment to the control group, investigators included a fairly even description of the treatments given to both experimental and control groups—a notable exception for both groups was a lack of specificity regarding the amount of time in each session devoted to the main components of the treatment. Additionally, many of the interventions were mail- or web-based and did not explicitly indicate the intervention setting. A comparison of the intensity of the treatments offered indicates that in some studies, the control group received “lighter” treatment doses than the experimental group (e.g., control group received a single pamphlet whereas the experimental group received tailored monthly magazines for 8 months [48], packet of printed booklets vs. highly interactive web-based program [49], manual vs manual coupled with coaching calls, tailored newsletters, and personalized feedback [56]). Many studies appeared comparable across a range of non-specific factors that could affect study outcomes [42, 51, 52]. One example of comparable treatment is Wolf et al. [57] who provided both experimental and control groups with a brochure (different topics) and tailored telephone education. Healy et al. [39] offers a second example in which both groups received a treatment that was the same length of time (7 50-min sessions over 1.5 weeks), used similar teaching strategies (i.e., lecture, discussion, question/answer, group activities), and differed only on content taught.

The 7 dismantling (additive) component active control studies tended to have 2 or more experimental groups. Interestingly, in all but one of these studies, the differences between the experimental and control treatments hinged on tailoring [61]. The control, or comparison, group in nearly all of these studies received less personalized and less intensive treatment than the experimental group [54, 59, 61, 63, 64]. In one study, for example, 3 groups of women either received non-tailored newsletters, tailored newsletters, or tailored newsletters and visits with lay health advisors [64]. Because of the derivative nature and increasing intensity of treatment provided by most dismantling studies [54, 59, 61, 63, 64], there was an imbalance in non-specific factors between/among study groups. The in-person and frequent phone contact received by one experimental group vs ongoing access to the project website and automated individual risk profiling given to a second experimental group vs printed materials provided one time to control participants demonstrated the imbalanced attention across study groups [59]. Among dismantling studies, the greatest balance in non-specific effects was achieved by Resnicow et al. [53] in that both experimental and control groups received the same newsletters except the tailoring of the experimental newsletters was more specific.

An additional two dismantling studies were classified as “mixed” [46, 65] because the control participants received an alternate treatment that was not a derivative of the experimental group but was similar to treatment provided to control participants in alternative active treatment conditions. For instance, control condition participants in one study received 2 45-min web sessions on anatomy whereas those in the 2 experimental groups received 2 45-min web sessions on nutrition or 2 45-min web sessions plus a 45-min booster session [65]. The comparability of treatment provided to control groups in these 2 mixed dismantling active control studies tended to be more balanced on non-specific factors than the other 7 dismantling studies that did not have an alternative treatment.

### Other findings

Reports of sample size and attrition were uneven. Some studies provided a complete description of total numbers recruited and retained, by treatment group, at each phase of the study [55, 67], with several including CONSORT diagrams [48, 53–55, 57–59, 63, 65, 66, 68, 69]. However, other studies only reported sample sizes at baseline [70], posttest [43, 71, 72], those completing both pretest and posttest [22, 45], or sample sizes and/or attrition rates for both groups combined [41, 73].

More than 3 out of 4 studies reviewed had random assignment of participants or intact groups (e.g., classrooms). Of the 10 non-randomized trials, half had no treatment control conditions. Of the remainder, one did not address randomization [41], one indicated the experimental group was comprised of students in classrooms with teachers who volunteered to participate [38], and another involving college students used intact classes and did not randomize the classes [44]. Two studies offered more explanation. One that was offered in WIC clinics indicated randomization was impractical and stated that “the practicality of being able to actually study comparisons of nutrition education intervention modalities in a typical clinic setting overcompensated for the lack in ability to develop a randomized design” [37],p.754. Authors of the second study offered this rationale, “The high cost and limited availability of randomized controlled trials in community settings highlight a need to evaluate and report on nonrandomized interventions that can be implemented in existing community settings” [45],p. 265.

Terminology used to describe control groups was not always consistent with definitions in Table 1. For example, two papers referred to control groups who received usual instruction as no treatment controls [37, 43]. Another provided an alternative active treatment, yet referred to it as a standard treatment [48]. Still another referred to the alternative active treatment control group as an attention placebo group [65]. A placebo should have no effect on a person, however because learning likely occurred in this and other alternate education-related control conditions, the term placebo does not accurately describe the control condition.

## Discussion

The goal of this study was to conduct a systematic review of control groups in nutrition education interventions and describe how control conditions are reported in peer-reviewed primary outcomes journal articles in comparison with experimental conditions. The findings of this systematic review indicate that the articles sampled focused on a wide array of controlled nutrition education intervention studies. Most addressed fruits and vegetables, fat intake, and healthy eating and tended to target school children as well as limited resource youth and families enrolled in government sponsored programs. Overall, descriptions of experimental conditions, regardless of type of active control condition, tended to be far more complete than descriptions of control conditions. Studies tended to report nearly all key factors (i.e., intervention content, delivery mode, material type, total duration, setting, individual session/interaction components [e.g.,, number, duration or length, frequency, content], standardization procedures, procedures for assessing fidelity of implementation, references for materials, theoretical underpinnings, and randomization) for the experimental condition. However, descriptions of the experimental group commonly lacked procedures for blinding and tailoring (except when the study was comparing differences in the effect of tailoring). In contrast, control conditions lacked descriptions of many key factors, with the most commonly omitted factors being individual sessions/interactions (e.g., number, duration, frequency, content of individual sessions), procedures for standardization, procedures for assessing implementation fidelity, blinding procedures, rationale for the type of control group selected, and references for instructional materials. Additionally, the factors that were reported for control conditions tended to be less explicit and included fewer details than provided for the experimental condition. In many cases, too little information was provided to determine the comparability of the control group vis-à-vis non-specific factors. Overall, the descriptions of both control and experimental group treatments became more complete as the type of active control became stronger and more complex; that is, alternative active treatments and dismantling studies provided the most detailed descriptions of the control group condition whereas usual or standard control conditions provided the least detail.

One-third of the 43 reviewed studies had inactive control conditions (i.e., no treatment or delayed treatment), a research design that is considered weak [7, 17]. The Food and Drug Administration instructs that a no-treatment control be used only when investigation outcomes are entirely objective and cannot be biased by lack of blinding [74]—although this advice is directed at drug trials, it can be reasonably applied to education trials using inactive controls. For instance, in one delayed treatment study, researchers stated that a lack of blinding among those teaching the educational intervention was problematic (i.e., they “generally did not like to be randomized to the control condition [22],p.31”). Failure to implement procedures to prevent differential treatment, commitment, and engagement of both experimental and control condition instructors has the potential to confound results [75]. Likely many researchers conducting the 43 reviewed studies had implemented appropriate blinding procedures for participants, instructors, and researchers; however descriptions of procedures for blinding and/or prevention of differential treatment were not reported in most studies.

Active control conditions, considered a stronger research design than inactive [7], were used in two-thirds of the reviewed studies. In this and other studies [76], usual treatment was considered an active control whereas some researchers categorize usual treatment as inactive (or passive) because it typically is not structurally equivalent on non-specific factors to the experimental condition [6, 7, 24, 32, 76]. All usual treatment conditions in reviewed studies offered control groups traditional or regular instruction that did not include content offered to the experimental group. As Street and Luoma point out, it usually is not possible to equalize all non-specific factors (particularly credibility and outcome expectations) when using education about an unrelated topic as the usual treatment [6]. The limited information about the usual treatment given to control participants negated the possibility of confidently affirming equivalency of intensity and structure of control and experimental treatments.

A hallmark of evidence building is replicability. Similar to findings by researchers in other fields [12, 26], none of the experimental and control group treatments were sufficiently detailed to permit replication of the nutrition education interventions studied. About half of the experimental treatment descriptions included a reference for the intervention materials and a third of the control treatment descriptions included this information; these materials may mitigate replication issues associated with missing information in the reviewed article. Another alternative is to contact authors to obtain intervention details. When Glasziou et al. contacted authors who published non-pharmacological medical treatment intervention outcomes, treatment descriptions improved significantly; however one-third of the studies they reviewed still had insufficient detail, in part because study authors did not respond despite repeated attempts or were unwilling to provide additional information [26].

Standardization and fidelity procedures are equally important for control and experimental conditions—without these procedures, either group may receive more or less than the research protocol intended which likely will confound outcomes [75, 77, 78]. The limited reporting of standardization procedures (e.g., use of manuals, standard operating procedures) and process evaluation activities in the reviewed studies, and noted by others in psychological therapy research [77], indicates that either reports are incomplete or these procedures were not implemented—neither of which are helpful when trying to weigh the value of the study outcomes and determine whether treatment groups received differential treatment from unblinded research staff.

Random assignment is considered critical to minimizing biases in trial outcomes and maximizing accuracy of analysis of intervention effects. One-quarter of the reviewed studies did not randomly assign participants, and likely suffered from at least some selection bias [79]. Compounding the lack of randomization is that many of these same studies did not address participant or researcher blinding and/or procedures for assessing intervention implementation fidelity, all of which impair internal validity [79].

Reporting sample size seems like a fairly straightforward task, regardless of how complex an intervention design may be. Indeed, CONSORT flow diagrams [80] make reporting changes in sample size at each stage of the study clear and easy to report. Yet, many of the studies reviewed lacked key sample size information, a phenomenon noted by others [81, 82]. In some cases, sample size was not declared in tables reporting data [38, 47, 51, 72].

It is interesting that so few articles provided a justification for the type of control condition used, especially given this is a conscious decision made during study planning. A systematic review of psychosocial interventions with substance use disorders also found studies gave little justification for control group choice or considerations for how this choice may have affected study outcomes [24].

The classic work of Campbell and Stanley identifies the Solomon 4 group design as offering the greatest internal and external validity checks [5]. This design includes these groups: experimental (pretest-intervention-posttest), no pretest experimental (intervention-posttest), control (pretest-posttest), no pretest control (posttest). Comparison of posttest scores across the 4 groups reveals whether changes are the result of the intervention and/or learning from the test [5, 79]. None of the reviewed studies had non-pretested comparison groups. This lack of control for testing may have important implications; indeed researchers note that repeated measurements may encourage control condition participants to reflect on behaviors and initiate the behavior targeted in the experimental condition [83, 84]. Another research group suggested that the research design for psychosocial treatments that most closely equates to a double-blind design is one that compares “two bona fide interventions … delivered by advocates for those interventions” [24],p. 426–427. In the reviewed studies, just one study met these criteria [57]. That is, Wolf et al. reported that immigrant men were given either a fruit/vegetable or prostate cancer prevention brochure [57]. Both groups received 2 tailored telephone education calls that could be considered to be delivered by an “advocate” because callers use a standardized telephone protocol and were audiotaped as a check for fidelity of delivery (however, no mention was made as to whether different callers were used for each treatment). Still another research group felt that to disentangle effects of the “active ingredient” from effects of non-specific factors, studies should include 3 groups: wait list control, attention control, and experimental group [85]. Many of the reviewed studies had 2 of these groups, but none had all 3.

Dismantling designs make it possible to separately account for the effects of each intervention component. However, the reviewed dismantling studies were mostly additive—that is, the treatment groups received increasingly intensive treatments thereby making it impossible to ascertain whether it was the greater dose of the additive treatment that contributed to changes or just the additional element [46, 54, 58, 63, 65]. For instance, one had a control group who received 12 weekly non-tailored newsletters by mail, an experimental group received 12 weekly tailored newsletters by mail, and another experimental group received the 12 weekly tailored newsletters plus weekly home visits from a promotora (lay health advisor/counselor) [64]. There was not a group who received only promotora visits, thus differentiating between intensity and independent effects of the promotora was not possible.

In the words of Montgomery et al., “[p]oor reporting limits the ability to replicate interventions, synthesise evidence in systematic reviews, and utilise findings for evidence-based policy and practice. The lack of guidance for reporting the specific methodological features of complex intervention RCTs contributes to poor reporting” [86],p.99. To improve reporting, the CONSORT extension underway for randomized controlled trials of social and psychological interventions may be appropriate and/or adaptable for health and nutrition education and promotion programs [86]. Methods for overcoming deficiencies in reporting design and execution of both control and experimental conditions reported by others may serve as models for reporting nutrition education interventions [7, 87]. One research group has even suggested creating a repository of treatment descriptions, citing the Centers for Disease Control and Prevention’s Replicating Effective Programs (https://www.cdc.gov/hiv/research/interventionresearch/rep/index.html) as an example, and establishing a detailed checklist of characteristics to be included in intervention descriptions [26]. In fact, the supplementary table published by Greaves and colleagues is an excellent reporting method that ensures all salient elements are included [87]. Table 3 in this paper is another tool for ensuring key information is reported in nutrition education outcomes papers.

Strengths of this review lie in the large number of papers included and the extensive extraction of data contributing to this comprehensive description of control groups in nutrition education interventions and how they and experimental conditions are recounted in peer-reviewed journals. Additionally, it is the first study to explore control conditions in nutrition education and is among the first in any field to examine this critically important research intervention study design and reporting component [7, 26, 85]. This study is, however, limited to studies conducted in the United States. Furthermore, the studies reviewed likely included at least some of the extracted factors reported as missing in Additional file 1: Table S5, but did not explicitly report them in the published paper. Also, no attempt was made to examine cited sources, which may supplement the information provided in the reviewed papers. Examination of the appropriateness of outcome measures, adequacy of sample sizes, and effect of control condition on study outcomes were beyond the scope of this review, but are important targets for future investigations.

## Conclusions

Calls for more transparency and detail in reporting interventions have occurred sporadically since at least 1991, yet little has changed [77, 88]. In this day and age of ever constricting research funding, coupled with the dire need for interventions that effectively improve nutritional status and associated outcomes, it is imperative that intervention research use more robust study designs that permit us to understand the effects of each component of the intervention [26, 85]. Additionally, researchers and journal editors should assume the responsibility for ensuring that practitioners can easily access the details needed to implement effective interventions with fidelity. The key historic barrier to reporting this data in printed form has been overcome with electronic publishing [26, 89]. Clearly there is a great deal of opportunity to improve intervention study design and reporting—seizing this opportunity can only help to advance the field and improve consumer health. A goal set at the outset of the investigation reported here is to open a dialogue among nutrition education researchers that leads to improved reporting of control and experimental condition treatments in intervention evaluation studies to promote advancement and impact of our work.

## Additional file

Additional file 1: Table S5. Factors extracted in systematic review of articles. (DOCX 37 kb)
