# Supplementary material for: Systematic review of control groups in nutrition education intervention research
Source: Int J Behav Nutr Phys Act. 2017 Jul 11;14:91. doi: 10.1186/s12966-017-0546-3 (PMC5504837; doi:10.1186/s12966-017-0546-3)
Supplement: Additional file 1: Table S5. — Factors extracted in systematic review of articles. (DOCX 37 kb) [file 12966_2017_546_MOESM1_ESM.docx]

**Table 5. Factors Extracted in Systematic Review of Articles, Part 1 of 7**

|  | **Inactive Control Condition** | | | | | | | |
| --- | --- | --- | --- | --- | --- | --- | --- | --- |
|  | **No Treatment Control** | | | | | | | |
| **Description of…** | **Powers et al, 2005 [71]** | **Kemirembe et al, 2011 [72]** | **Katz et al, 2011 [70]** | **Keihner et al, 2011 [96]** | **Backman et al, 2011 [67]** | **Roofe et al, 2011 [97]** | **McCarthy et al, 2012 [98]** | **Alaimo et al, 2015 [69]** |
|  | **E#** | **E** | **E** | **E** | **E** | **E** | **E** | **E** |
| 1. ... overall intervention content | X | X | X | X | X | X | X |  |
| 2. ...how intervention was tailored to participants |  |  |  | X | X |  | X |  |
| 3. ...intervention delivery mode | X | X | X | X | X | X | X | X |
| 4. ...intervention material type used to provide content | X | X | X | X | X | X | X | X |
| 5. ...total duration of intervention | X | X | X | X | X | X | X | X |
| 6. ...intervention setting | X | X | X | X | X | X | X | X |
| 7. ...individual sessions/interactions |  |  |  |  |  |  |  |  |
| a. number of individual sessions or interactions | X | X | X | X | X | X* | X | X |
| b. duration of individual sessions/interactions or length of materials |  | X | X | X | X | X* |  |  |
| c. frequency of individual sessions/interactions | X | X |  |  | X |  | X |  |
| d. content of each session/interaction | X |  | X | X | X | X* | X |  |
| e. duration of each main component of each individual session/interaction |  |  |  | X |  |  |  |  |
| 8. ...procedures for standardization | X |  |  | X | X | X |  | X |
| 9. ...procedures for assessing intervention implementation with fidelity |  |  |  | X | X |  |  | X |
| 10.  ... procedures for blinding participant, instructor, and/or researcher |  |  |  |  |  |  |  |  |
| 11.  …rationale for selection of control group type |  |  |  |  |  |  |  |  |
| Reference Provided for Instructional Materials | X |  |  |  |  |  |  |  |
| Theoretical Underpinning of Intervention# | SCT |  |  | SCT, RT | SCT |  | SCT |  |
| Randomization of Participants or Groups |  |  | X | X | X |  |  |  |

**Table 5. Factors Extracted in Systematic Review of Articles, Part 2 of 7**

|  | **Inactive Control Condition** | | | | | | |
| --- | --- | --- | --- | --- | --- | --- | --- |
|  | **Wait-List Delayed Control** | | | | | | |
| **Description of…** | **Townsend et al, 2008 [22]** | **Eicher-Miller, et al, 2009 [99]** | **Wilcox et al, 2013 [68]** | **Bogart et al, 2014 [73]** | **Dollahite et al, 2014 [62]** | **Kattelmann et al, 2014 [66]** | **Madsen et al, 2015 [100]** |
|  | **E#** | **E** | **E** | **E** | **E** | **E** | **E** |
| 1. ... overall intervention content | X | X | X | X | X | X | X |
| 2. ...how intervention was tailored to participants |  | X | X |  | X | X |  |
| 3. ...intervention delivery mode | X | X | X | X | X | X | X |
| 4. ...intervention material type used to provide content | X | X | X | X | X | X | X |
| 5. ...total duration of intervention | X | X | X | X | X | X | X |
| 6. ...intervention setting | X | X | X | X | X | X* | X |
| 7. ...individual sessions/interactions |  |  |  |  |  |  |  |
| a. number of individual sessions or interactions | X | X |  | X | X | X |  |
| b. duration of individual sessions/interactions or length of materials | X | X |  |  |  |  |  |
| c. frequency of individual sessions/interactions |  | X |  | X | X | X |  |
| d. content of each session/interaction |  | X |  | X | X |  |  |
| e. duration of each main component of each individual session/interaction |  |  |  |  |  |  |  |
| 8. ...procedures for standardization | X | X | X | X | X | X | X |
| 9. ...procedures for assessing intervention implementation with fidelity | X | X | X | X |  |  |  |
| 10.  ... procedures for blinding participant, instructor, and/or researcher |  | X |  | X |  |  |  |
| 11.  …rationale for selection of control group type |  |  |  |  | X |  |  |
| Reference Provided for Instructional Materials | X |  |  |  | X |  |  |
| Theoretical Underpinning of Intervention# |  | SCT | CBPR, SEM, SCT | SCT. CBPR, DI | ALT | P-P, TM |  |
| Randomization of Participants or Groups | X | X | X | X | X | X | X |

**Table 5. Factors Extracted in Systematic Review of Articles, Part 3 of 7**

|  | **Active Control Condition** | | | | | | | | | | | | | |
| --- | --- | --- | --- | --- | --- | --- | --- | --- | --- | --- | --- | --- | --- | --- |
|  | **Usual or Standard Treatment** | | | | | | | | | | | | | |
| **Description of…** | **Hopper et al, 2005 [47]** | | **Pobocik et al., 2009 [41]** | | **Dzewaltowski et al, 2010 [55]** | | **Bensley et al, 2011 [37]** | | **McCaughtry et al, 2011 [43]** | | **Wall et al, 2012 [40]** | | **Herbert et al, 2013 [38]** | |
|  | E | C | E | C | E | C | E | C | E | C | E | C | E | C |
| 1. ... overall intervention content | X | X | X | X | X |  | X | X | X | X | X | X | X |  |
| 2. ...how intervention was tailored to participants |  |  |  |  |  |  | X |  |  |  |  |  |  |  |
| 3. ...intervention delivery mode | X | X* | X | X* | X | X* | X | X | X* | X | X* |  | X | X* |
| 4. ...intervention material type used to provide content | X |  | X |  | X |  | X | X | X |  | X |  | X |  |
| 5. ...total duration of intervention | X |  | X |  | X |  |  |  | X | X | X |  | X |  |
| 6. ...intervention setting | X | X | X | X | X | X | X | X | X | X | X | X | X | X |
| 7. ...individual sessions/interactions |  |  |  |  |  |  |  |  |  |  |  |  |  |  |
| a. number of individual sessions or interactions | X |  | X |  | X |  | X | X | X |  | X |  | X |  |
| b. duration of individual sessions/interactions or length of materials | X |  | X |  | X |  |  |  | X |  |  |  | X |  |
| c. frequency of individual sessions/interactions | X |  | X |  | X |  | X | X | X |  | X |  | X |  |
| d. content of each session/interaction |  |  | X |  |  |  | X | X |  |  | X |  | X |  |
| e. duration of each main component of each individual session/interaction |  |  | X |  |  |  |  |  |  |  |  |  | X |  |
| 8. ...procedures for standardization | X |  | X |  | X |  | X |  | X |  | X |  | X |  |
| 9. ...procedures for assessing intervention implementation with fidelity | X |  |  |  | X | X |  |  | X | X |  |  | X |  |
| 10.  ... procedures for blinding participant, instructor, and/or researcher |  |  |  |  | @ |  |  |  | X | X |  |  |  |  |
| 11.  …rationale for selection of control group type |  |  |  |  |  |  |  |  |  |  |  |  |  |  |
| Reference Provided for Instructional Materials | X |  | X |  | X |  | X |  | X |  | X |  |  |  |
| Theoretical Underpinning of Intervention# | SLT |  | SCT |  | SCT, EDSA | | TM |  | CT |  |  |  | SCT, TRA, HBM | |
| Randomization of Participants or Groups | X |  |  |  | X |  |  |  | X |  | X |  |  |  |

**Table 5. Factors Extracted in Systematic Review of Articles, Part 4 of 7**

|  | **Active Control Condition** | | | | | | | | | | | | | |
| --- | --- | --- | --- | --- | --- | --- | --- | --- | --- | --- | --- | --- | --- | --- |
|  | **Alternative Active Treatment** | | | | | | | | | | | | | |
| **Description of…** | **Devine et al, 2005 [45]** | | **Nitzke et al, 2006 [48]** | | **Mitchell et al, 2006 [52]** | | **McCarthy et al, 2007 [51]** | | **Cook et al, 2007 [49]** | | **Greene et al, 2008 [56]** | | **Wolf et al, 2009 [57]** | |
|  | E | C | E | C | E | C | E | C | E | C | E | C | E | C |
| 1. ... overall intervention content | X | X | X | X | X | X | X | X | X | X | X | X | X | X |
| 2. ...how intervention was tailored to participants | X |  | X | N/A | X | X | X | X | X | X | X |  | X | X |
| 3. ...intervention delivery mode | X | X* | X | X | X | X | X | X | X | X | X | X | X | X |
| 4. ...intervention material type used to provide content | X |  | X | X | X | X | X | X | X | X | X | X | X | X |
| 5. ...total duration of intervention | X | X | X | X | X | X | X | X | X | X | X | X | X | X |
| 6. ...intervention setting | X | X | X* | X* | X | X | X | X | X* | X* | X* | X* | X* | X* |
| 7. ...individual sessions/interactions |  |  |  |  |  |  |  |  |  |  |  |  |  |  |
| a. number of individual sessions or interactions | X |  | X | X | X | X | X | X | X | X | X |  | X | X |
| b. duration of individual sessions/interactions or length of materials | X |  |  |  |  |  | X | X |  | X |  |  | X | X |
| c. frequency of individual sessions/interactions | X |  | X | X | X | X | X | X | X |  | X |  | X | X |
| d. content of each session/interaction | X |  | X | X | X | X | X |  | X | X | X |  | X | X |
| e. duration of each main component of each individual session/interaction | X |  |  |  |  |  |  |  |  |  |  |  |  |  |
| 8. ...procedures for standardization | X |  | X |  | X | X |  | X | X | X | X | X | X | X |
| 9. ...procedures for assessing intervention implementation with fidelity |  |  |  |  | X | X |  |  |  |  |  |  | X | X |
| 10.  ... procedures for blinding participant, instructor, and/or researcher |  |  |  |  | X | X | X | X |  |  |  |  | X | X |
| 11.  …rationale for selection of control group type |  |  |  |  |  |  |  |  | X | X |  |  |  |  |
| Reference Provided for Instructional Materials | X | X | X | X |  |  | X | X |  |  | X | X | X | X |
| Theoretical Underpinning of Intervention# |  |  | TM |  | SCT |  | SLT, SAT, SEM | | SCT, TM | | TM | | HBM, TM | |
| Randomization of Participants or Groups |  |  | X |  | X |  | X |  | X |  | X |  | X |  |

**Table 5. Factors Extracted in Systematic Review of Articles, Part 5 of 7**

|  | **Active Control Condition** | | | | | | | | | |
| --- | --- | --- | --- | --- | --- | --- | --- | --- | --- | --- |
|  | **Alternative Active Treatment, Cont’d** | | | | | | | | | |
| **Description of…** | **Clifford, et al, 2009 [50]** | | **Hekler, et al, 2010 [44]** | | **Glanz et al, 2012 [60]** | | **McClelland et al, 2013 [42]** | | **Healy et al, 2015 [39]** | |
|  | E | C | E | C | E | C | E | C | E | C |
| 1. ... overall intervention content | X | X | X | X | X | X | X | X | X | X |
| 2. ...how intervention was tailored to participants | X |  |  |  |  |  |  |  |  |  |
| 3. ...intervention delivery mode | X | X | X | X | X | X | X | X | X | X |
| 4. ...intervention material type used to provide content | X | X | X |  | X | X | X | X | X | X |
| 5. ...total duration of intervention | X | X | X | X | X | X | X | X | X | X |
| 6. ...intervention setting | X* | X* | X | X |  |  | X | X | X | X |
| 7. ...individual sessions/interactions |  |  |  |  |  |  |  |  |  |  |
| a. number of individual sessions or interactions | X | X |  |  | X | X | X | X | X | X |
| b. duration of individual sessions/interactions or length of materials | X | X |  |  | X | X |  |  | X | X |
| c. frequency of individual sessions/interactions | X | X |  |  | X | X | X | X | X | X |
| d. content of each session/interaction | X |  |  |  | X | X | X |  |  | X |
| e. duration of each main component of each individual session/interaction |  |  |  |  |  |  |  |  |  |  |
| 8. ...procedures for standardization | X | X |  |  |  |  |  |  | N/A | N/A |
| 9. ...procedures for assessing intervention implementation with fidelity | X | X |  |  |  |  |  |  |  |  |
| 10.  ... procedures for blinding participant, instructor, and/or researcher |  |  |  |  |  |  | X | X | N/A |  |
| 11.  …rationale for selection of control group type |  |  | X | X | X | X |  |  |  |  |
| Reference Provided for Instructional Materials |  |  |  |  |  | X | X | X |  | X |
| Theoretical Underpinning of Intervention# | SCT |  |  |  | SCT, TPB, CIP | | HBM, TM, TPB | |  |  |
| Randomization of Participants or Groups | X |  |  |  | X |  | X |  | X |  |

**Table 5. Factors Extracted in Systematic Review of Articles, Part 6 of 7**

|  | **Active Control Condition** | | | | | | | | | | | | | | | |
| --- | --- | --- | --- | --- | --- | --- | --- | --- | --- | --- | --- | --- | --- | --- | --- | --- |
|  | **Dismantling (or Additive) Component Active Treatment** | | | | | | | | | | | | | | | |
| **Description of…** | **Elder et al, 2009 [64]** | | | **Resnicow et al, 2009 [53]** | | **Gans et al, 2009 [58]** | | | | **Alexander, et al, 2010 [63]** | | | | **Hughes et al, 2011 [59]** | | |
|  | E1 | E2 | C | E | C | E1 | E2 | E3 | C | | E1 | E2 | C | E1 | E2 | C |
| 1. ... overall intervention content | X | X | X | X | X | X | X | X | X | | X | X | X | X | X | X |
| 2. ...how intervention was tailored to participants | X | X | N/A | X | X | X | X | X | N/A | | X | X | N/A | X | X |  |
| 3. ...intervention delivery mode | X | X | X | X | X | X | X | X | X | | X | X | X | X | X | X |
| 4. ...intervention material type used to provide content | X | X | X | X | X | X | X | X | X | | X | X | X | X | X | X |
| 5. ...total duration of intervention | X | X | X | X | X | X | X | X | X | | X | X | X | X | X | X |
| 6. ...intervention setting | X | X | X | X* | X* | X* | X* | X* | X* | | X* | X* | X* | X* | X* | X |
| 7. ...individual sessions/interactions |  |  |  |  |  |  |  |  |  | |  |  |  |  |  |  |
| a. number of individual sessions or interactions | X | X | X | X | X | X | X | X | X | | X | X | X | X | X | X |
| b. duration of individual sessions/interactions or length of materials | X | X |  | X | X |  |  |  | X | | X | X | X |  |  |  |
| c. frequency of individual sessions/interactions |  |  | X | X | X | X | X | X | X | | X | X | X |  | X | X |
| d. content of each session/interaction | X | X |  | X | X | X | X | X | X | |  |  |  |  |  | X |
| e. duration of each main component of each individual session/interaction | X | X |  |  |  |  |  |  |  | |  |  |  |  |  |  |
| 8. ...procedures for standardization | X |  | X | X | X |  |  |  |  | | X | X | X |  | X | X |
| 9. ...procedures for assessing intervention implementation with fidelity | X | X |  | X | X | X | X | X |  | |  |  |  |  | X |  |
| 10.  ... procedures for blinding participant, instructor, and/or researcher | X | X | X |  |  | X | X | X | X | | X | X | X | X | X | X |
| 11.  …rationale for selection of control group type |  |  |  |  |  |  |  |  |  | |  |  |  |  |  |  |
| Reference Provided for Instructional Materials |  |  |  |  |  | X | X | X |  | |  |  |  | X | X |  |
| Theoretical Underpinning of Intervention# | SCT, CPM, SSSI | | | BIT |  | TM, SCT | | | | | SCT, TM, HBM | | | TM |  |  |
| Randomization of Participants or Groups | X |  |  | X |  | X |  |  |  | | X |  |  | X |  |  |

**Table 5. Factors Extracted in Systematic Review of Articles, Part 7 of 7**

|  | **Active Control Condition** | | | | | | | | | | |
| --- | --- | --- | --- | --- | --- | --- | --- | --- | --- | --- | --- |
|  | **Dismantling (or Additive) Component Active Treatment** | | | | | **Mixed: Dismantling (or Additive) Component, Active Control & Alternative Active Treatment** | | | | | |
| **Description of…** | **Ratcliffe et al, 2011 [61]** | | **Gans et al, 2015 [54]** | | | **Franko et al, 2008 [65]** | | | **Ievers-Landis et al, 2005 [46]** | | |
|  | E | C | E1 | E2 | C | E1 | E2 | C | E1 | E2 | C |
| 1. ... overall intervention content | X | X | X | X | X | X | X | X | X | X | X |
| 2. ...how intervention was tailored to participants |  |  | X | X | N/A |  |  |  |  |  |  |
| 3. ...intervention delivery mode | X | X | X | X | X | X | X | X | X | X | X |
| 4. ...intervention material type used to provide content |  |  | X | X | X | X | X | X | X | X | X |
| 5. ...total duration of intervention | X | X | X | X | X | X | X | X | X | X | X |
| 6. ...intervention setting | X | X | X* | X* | X* | X | X | X | X | X | X |
| 7. ...individual sessions/interactions |  |  |  |  |  |  |  |  |  |  |  |
| a. number of individual sessions or interactions | X |  | X | X | X | X | X | X | X | X | X |
| b. duration of individual sessions/interactions or length of materials | X |  |  | X |  | X | X | X | X | X | X |
| c. frequency of individual sessions/interactions | X |  | X | X |  | X | X | X | X | X | X |
| d. content of each session/interaction |  |  | X | X |  |  |  |  | X | X | X |
| e. duration of each main component of each individual session/interaction | X |  |  |  |  |  |  |  |  |  |  |
| 8. ...procedures for standardization |  |  | X | X |  | X | X | X | X | X | X |
| 9. ...procedures for assessing intervention implementation with fidelity |  |  | X | X | X | X | X | X | X | X | X |
| 10.  ... procedures for blinding participant, instructor, and/or researcher |  |  |  |  |  |  |  |  | X | X | X |
| 11.  …rationale for selection of control group type |  |  |  |  |  | X |  |  | X | X | X |
| Reference Provided for Instructional Materials |  |  |  |  |  | X | X |  |  |  |  |
| Theoretical Underpinning of Intervention# | SCT |  |  |  |  |  |  |  | SCT |  |  |
| Randomization of Participants or Groups | X |  | X |  |  | X |  |  | X |  |  |

* Implied

@Stated researchers not blinded

**# Abbreviations Used:**

E= Experimental Group

C=Control Group

X=Element reported in paper

N/A= Not applicable

SCT=Social Cognitive Theory

RT=Resiliency Theory

SEM=Social Ecological Model

CBPR=Community-based Participatory Research

ALT=Adult Learning Theory

P-P=Precede Procede

TM=Transtheoretical Model

SLC=Social Learning Theory

CT=Constructivist theory

HBM=Health Belief Model

TRA=Theory of Reasoned Action

SAT=Social Action Theory

CIP=Conscious Information Processing Theory

TPB=Theory of Planned Behavior

CPM=Communication Persuasion Model

SSSI=Social Support and Social Influence Theories

BIT=Black Identity Theory

EDSA=Ecological Developmental Systems Approach

DI=Diffusion of Innovations Theory
